# Monocyte-Induced Prostate Cancer Cell Invasion is Mediated by Chemokine ligand 2 and Nuclear Factor-κB Activity

**DOI:** 10.4172/2155-9899.1000308

**Published:** 2015-04-05

**Authors:** Paul F Lindholm, Neela Sivapurapu, Borko Jovanovic, André Kajdacsy-Balla

**Affiliations:** 1 Department of Pathology, Northwestern University, The Feinberg School of Medicine, Chicago, USA; 2 Indigenèse Biotechnologies, Hyderabad, India; 3 Department of Preventive Medicine and Bioinformatics Core, Northwestern University, The Feinberg School of Medicine, Chicago, USA; 4 Department of Pathology, University of Illinois at Chicago College of Medicine, Chicago, USA

**Keywords:** Inflammation, Co-culture, Paracrine, MCP-1, NF-κB

## Abstract

**Study Background:**

The tumor microenvironment contains inflammatory cells which can influence cancer growth and progression; however the mediators of these effects vary with different cancer types. The mechanisms by which prostate cancer cells communicate with monocytes to promote cancer progression are incompletely understood. This study tested prostate cancer cell and monocyte interactions that lead to increased prostate cancer cell invasion.

**Methods:**

We analyzed the prostate cancer cell invasion and NF-κB activity and cytokine expression during interaction with monocyte-lineage cells in co-cultures. The roles of monocyte chemotactic factor (MCP-1/CCL2) and NF-κB activity for co-culture induced prostate cancer invasion were tested. Clinical prostate cancer NF-κB expression was analyzed by immunohistochemistry.

**Results:**

In co-cultures of prostate cancer cell lines with monocyte-lineage cells, (C-C motif) ligand 2 (CCL2) levels were significantly increased when compared with monocytes or cancer cells cultured alone. Prostate cancer cell invasion was induced by recombinant CCL2 in a dose dependent manner, similar to co-cultures with monocytes. The monocyte-induced prostate cancer cell invasion was inhibited by CCL2 neutralizing antibodies and by the CCR2 inhibitor, RS102895. Prostate cancer cell invasion and CCL2 expression induced in the co-cultures was inhibited by Lactacystin and Bay11-7082 NF-κB inhibitors. Prostate cancer cell NF-κB DNA binding activity depended on CCL2 dose and was inhibited by CCL2 neutralizing antibodies. Clinical prostate cancer NF-κB expression correlated with tumor grade.

**Conclusions:**

Co-cultures with monocyte-lineage cell lines stimulated increased prostate cancer cell invasion through increased CCL2 expression and increased prostate cancer cell NF-κB activity. CCL2 and NF-κB may be useful therapeutic targets to interfere with inflammation-induced prostate cancer invasion.

## Introduction

Prostate cancer is the most common malignancy in American men and metastases are responsible for most prostate cancer mortality. Cancer metastasis is a multistep process in which the tumor microenvironment plays a role to promote aggressive cancer cell behavior [1,2]. Inflammatory stimuli, especially involving macrophages and their accompanying cytokines are increasingly recognized factors that can promote cancer progression, but how this occurs is not fully understood [1-6].

Tumor-associated macrophages (TAM) and stromal cells may support tumor progression by promoting angiogenesis, immune suppression or direct effects on tumor cells. Co-cultures of breast cancer cells and monocytes have been shown to express cell-secreted factors which cause paracrine stimulation of tumor growth and progression [7-10]. Several tumor specific cell-secreted factors have been identified that mediate interactions between cancer cells and monocytes [8-13]. Paracrine stimulation of prostate cancer cells and monocytes has been hypothesized; however, studies are needed to determine precisely how prostate cancer cells and monocytes cross-communicate to promote prostate cancer growth and progression [14,15].

Several cytokines and chemokines are produced by macrophages in the tumor microenvironment including IL-8, stromal-derived factor-1 (SDF-1) and CCL2 [16-18]. Prostate cancer cells express receptors for these and other chemokines and can respond to stimulation with growth, proliferation and metastasis [19,20]. Interleukin 8 produced at high levels by prostate cancer cells can promote angiogenesis and androgen independent tumor growth [16]. Prostate cancer cells that express CCL2 have been shown to cause monocyte and osteoclast recruitment with resulting cancer cell growth and survival [21,22]. Prostate cancer proliferation and metastasis may also be stimulated by SDF-1 (CXCL12), CCL2 and other factors [17,19,22-24]. These cytokines may be involved in cross-communication of prostate cancer and inflammatory cells to stimulate cancer cell gene expression, survival and invasion [25-27].

Stimulation of prostate cancer cell growth and metastasis by cytokines including TNF-α, GRO-α and RANK ligand are dependent on signaling events leading to NF-κB activation [28-30]. Previous studies have shown the necessary role of NF-κB transcription factor activity for prostate cancer cell invasion and metastasis [31-33]. NF-κB activity has also been shown to be essential for activation of cytokine and extracellular protease expression necessary for prostate cancer invasion and metastasis [30,34,35]. However, the role of NF-κB in monocyte-induced prostate cancer cell invasion has not been determined. The purpose of this study was to identify factors involved in cross-communication between prostate cancer cells and monocytes mediating increased prostate cancer cell invasion.

In this study, co-cultures of prostate cancer cells and monocytes showed greatly increased CCL2 levels associated with increased prostate cancer cell invasion. Co-cultures with monocytes also showed that CCL2 expression and prostate cancer cell NF-κB activity were required for monocyte-induced prostate cancer cell invasion. This study explored the role of CCL2 and NF-κB activity and indicates that these factors may be key molecular targets to inhibit inflammation-associated prostate cancer progression.

## Materials and Methods

### Cell cultures

Human prostate cancer cells PC-3, LNCaP, DU145 and monocytoid U-937 and THP-1 cell lines were purchased from ATCC, Rockville, Maryland. The PC-3 High and Low Invasive cell lines were selected by three serial passages through Matrigel reconstituted basement membranes (Becton Dickinson, Lincoln Park, NJ) in a Transwell chamber with 8 μM pore size [31]. The selected cells were placed in co-cultures with monocyte-lineage U-937 or THP-1 cells at standard seeding densities. For transfection experiments, the prostate cancer cells were exposed to 5 μg of dominant negative pEGFP-IκBα S32/S36 expression vector or control vector pEGFP-C1 (Clontech, Mountain View, CA) [32]. All cells were maintained in a humidified atmosphere of 5% CO_2_ at 37°C in RPMI 1640 medium supplemented with 10% fetal bovine serum (FBS, Biofluids, Rockville, MD); 2 mM Lglutamine; 100 units/mL penicillin and 100 μg/mL streptomycin (Life Technologies, Inc.). Bay11-7082 and RS102895 HCl were obtained from Sigma-Aldrich, St. Louis, MO. Lactacystin was purchased from Biomol (Enzo Life Sciences, Inc.), Plymouth Meeting, PA. Gefitinib (N-(3-chloro-4-fluoro-phenyl)-7-methoxy-6-(3-morpholin-4-ylpropoxy)quinazolin-4-amine) was purchased from Tocris Bioscience. Recombinant human CCL2 (279-MC), anti-human CCL2 neutralizing antibodies (MAB279) and IL-8 (208-IL) were purchased from R&D Systems, Inc., Minneapolis, MN.

### Invasion assay

The invasion assay was performed by adding 50,000 [^3^H] Thymidine (GE Healthcare Bio-Sciences, Piscataway, NJ) pulse-labeled cells to the upper chamber coated with 35 μg Matrigel (Becton Dickinson, Lincoln Park, NJ) separated from the lower chamber by 8 μm pores in Transwell chamber plates (Costar, Corning, NY) [32]. The effect of human monocytes on prostate cancer cell invasion was tested using Matrigel-coated Transwell chambers as previously described [31,32,36,37]. The cancer cell invasion assay was cultured with or without 10,000 U-937 or THP-1 monocytoid cell lines (ATCC) or 20,000 human peripheral blood monocytes added to the lower chamber. The cells were cultured in RPMI 1640 medium supplemented with 10% fetal bovine serum [31,36]. The cell invasion assays were tested in triplicate and each experiment was performed 3 or more times. In this assay, the ^3^H-thymidine labeled cancer cells that passed through the Matrigel membrane into the lower Transwell chamber were counted and compared to the total number of labeled cells. The percentage of invaded cells in the lower chamber was determined by multiplying by 100, the cell associated ^3^H-thymidine cpm (recovered from each lower chamber by trypsinization) divided by the total cell-associated cpm initially added to each chamber. The relative percent invasion was normalized to that of PC-3 High Invasion cells not receiving co-culture stimulation with monocytes. The percent invasion was not affected by cell proliferation or viability as determined by viable cell counts in the MTT assay [32].

### Cytokine array

The cytokine arrays were performed with RayBiotech human cytokine antibody array 3 as described in the manufacturer's protocol. Briefly, the cell culture supernatants from prostate cancer cells, monocyte-lineage cells and co-cultures were obtain at 48 hours under similar conditions to the invasion assays. The membranes were preincubated with 2 mL of 1× blocking buffer at room temperature for 30 minutes. The cultures contained serum-containing media, so an uncultured media aliquot was used as a negative control sample. The cytokine array membranes were completely covered and incubated with 1 mL of undiluted conditioned medium at for 2 hours at room temperature with gentle rotation and making sure to cover the membrane to prevent evaporation. The membranes were then incubated 2 times with 2 mL of 1× wash buffer at room temperature with gentle shaking. The membranes were incubated with a working solution of primary antibody at room temperature for 1-2 hours followed by washing steps. The membranes were then incubated with diluted HRP-conjugated streptavidin in 1× blocking buffer at room temperature for 2 hours followed by washing steps. The membranes were then incubated with detection reagent at room temperature for 2 minutes. The membranes were gently drained and sandwiched in between plastic sheets and exposed to Kodak X-Omat X-ray film in the darkroom. The film was initially exposed to the membranes for 40 seconds followed by re-exposing the film to the membranes depending on the intensity of signals. The relative expression levels of cytokines were made by comparing the signal intensities quantified by densitometry. The positive and negative controls were used to insure equal comparison of the membranes.

### ELISA

Extracts were made from the cancer cells grown to 70-80% confluence by incubating with cell extraction buffer containing protease inhibitor cocktail at 4°C.

The IL-6, IL-8, Gro-α and CCL2 ELISA assay kits were obtained from eBioscience, Inc. San Diego, CA. The cytokine ELISA was performed per manufacturer's instructions. Briefly, the cell supernatants or cell extracts were incubated in the assay plate which was pre-coated with the appropriate capture antibody. The samples were added in duplicate and incubated at room temperature (24°C) for 3 hours. The wells were washed 5 times and after the last wash, the wells were incubated with anti-CCL2 detection antibody diluted 1:250 for 1 hour. The wells were washed 5 times and then incubated with Avidin-horseradish peroxidase diluted 1:250 for 1 hour. The wells were washed and incubated with 100 μL of TMB (3,3′,5,5′-tetramethylbenzidine) substrate solution at room temperature for 10 minutes. The color development on the plate was monitored and the substrate reaction stopped by adding 100 μL of 2N H_2_SO_4_ stop solution into each well. The absorbance was read within 30 minutes at 450 nm on a Biotek Synergy H4 hybrid multi-mode microplate reader.

### NF-κB DNA binding assay

Cell extracts were prepared by microextraction [31]. Chemiluminescent NF-κB p65 Transcription Factor Assay Kit (Thermo Scientific Pierce, Rockford, lL). Briefly, the prostate cancer cell nuclear extracts were incubated in the assay plate coated with NF-κB oligonucleotides with binding buffer for 1 hour with mild agitation. The plate was then washed three times, followed by addition of 100 μL of anti p65 primary antibody. The plate was incubated for 1 hour with the primary antibody without agitation and was again washed followed by incubation with diluted secondary antibody for 1 hour without incubation. The plate was washed again, followed by incubation with 100 μL of chemiluminescent substrate. The chemiluminescence signals were read using a Biotek Synergy H4 hybrid multi-mode microplate reader within 10 minutes of adding the chemiluminescent substrate. Positive control wells were also incubated with wild-type and mutant oligonucleotides to establish the specificity of the NF-κB DNA binding signals. The results are reported as NF-κB luminescence signal units. To determine the NF-κB activity of PC-3 cells following transfection with pCMV 4-3 HA IκBα S32/36A or pCMV 4-3 HA control vector, NF-κB luciferase activity and electrophoretic mobility shift assays (EMSA) were used as previously described [31].

### Cell proliferation and viability assays

The Vybrant MTT cell proliferation assay kit (Molecular Probes, Eugene, OR) was used to measure cell proliferation as previously described [32,37]. To create a standard curve for viable cell number, the MTT signal was determined in parallel for viable prostate cancer cells seeded from 10,000 to 80,000 viable cells per well in a 96 well plate. The MTT signals were plotted against cell counts and viable cell number. The cancer cells were cultured in four wells and the viable cancer cell counts were determined from the standard curve. Parallel determinations of cell number and viability were made by counting cells on a hemocytometer slide using the trypan blue exclusion technique.

### Immunohistochemistry

Benign and cancer prostate tissues from Northwestern University and Robert H. Lurie Pathology Core facilities were assembled on tissue microarrays to represent prostate cancer grades and stages as well as samples of benign prostate. TMAs were obtained for research use after IRB review and approval. The TMAs were immunostained with antibodies to NF-κB subunits p50 (sc-114); p52 (sc-298); c-Rel (scsc-6955) from Santa Cruz Biotechnology; NF-κB p65 IgG2a from Zymed Biotechnology; CD68 (IgG3) (Dako clone PG-M1); CD-206 (Mouse IgG1, κ clone 15-2) (Biolegend) and CCL2 (R&D Systems, Minneapolis, MN). Each primary antibody was tested for optimal reactivity with serial dilutions following antigen retrieval. The antibodies were used at the following dilutions: anti p50 (1:40); anti p52 (1:100); anti c-Rel (1:100); anti p65 (1:200); anti CD68 (1:100); anti CCL2 (1:100) and anti CD206 (1:100). Manual NF-κB subunit immunostaining intensity scoring was performed independently by three pathologists. The nuclear immunostaining intensity for NF-κB subunits was graded using the following scoring criteria: 0 – negative; 1+, weak positive; 2+, intermediate positive, and 3+, strong positive [38,39]. The NF-κB subunit scores were then derived from the average NF-κB subunit immunostaining intensity times the percent positive nuclei for each tissue sample.

### Statistical analysis

Results are expressed with mean ± standard deviation. Statistical analysis was performed using GraphPad Prism version 3.00 for Windows, GraphPad Software, San Diego, California USA. The non-parametric Mann-Whitney test was used to compare prostate NF-κB subunit immunostaining intensity between groups. The Student's *t*-test was used for comparisons of the invasion assays between treatment groups. Significant differences were considered when P<0.05.

## Results

### Prostate cancer cells increase invasion when co-cultured with monocyte-lineage cells

**Figure 1:**
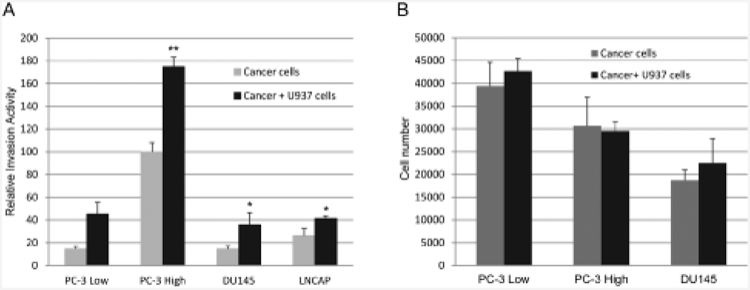
Effect of U-937 monocyte lineage cell co-cultures on prostate cancer cell invasion activity. (A) Relative invasion activity of labeled PC-3, DU145 or LNCaP cells with or without U-937 cells added to the lower chamber of the Transwell apparatus. The prostate cancer cell lines co-cultured with U-937 cells lead to significantly increased prostate cancer cell invasion when compared to the cancer cells cultured alone. The results are expressed as relative invasion activity at 48 hours of at least 3 independent experiments ± SD. *P<0.05; **P<0.001. (B) Viable prostate cancer cell number was determined by MTT assay after 48 hours of co-culture. The viable cancer cell numbers did not differ between cancer cells cultured alone and cancer cells co-cultured with U-937 cells. The data are representative of three independent experiments.

PC-3, DU145 and LNCaP Prostate cancer cells showed significantly increased invasion activity in co-cultures with U-937 cells above controls (Figure 1A). PC-3 cell invasion was similarly increased in co-cultures with THP-1 monocyte-lineage cells compared with PC-3 High invasion cells alone [40]. The co-cultures with U-937 cells did not significantly affect the viable prostate cancer cell number as determined by MTT assay (Figure 1B). Although the co-cultures caused increased prostate cancer cell invasion, this effect did not result from changes in viable cell number. The PC-3 Highly Invasive variant cells [31] were subsequently used to identify factors leading to increased invasion in the co-cultures with monocytes.

### Cytokines are differentially expressed in the prostate cancer/monocyte co-cultures

**Figure 2:**
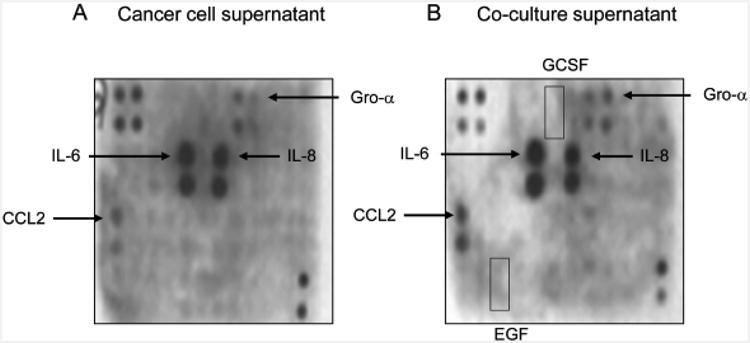
PC-3 and co-culture supernatant cytokine expression. (A) Cytokine array membrane was probed with conditioned media from PC-3 High Invasion variant cancer cells alone. (B) A Cytokine array membrane was probed with conditioned media from PC-3 High Invasion cells co-cultured with the U-937 cells. The co-culture supernatant cytokine array showed increased signals for monocyte chemo attractant protein-1 (CCL2) and Gro-alpha when compared with PC-3 High Invasion only supernatants. The control signals in the upper left and lower right corners were performed to ensure equal sample loading. The arrays are representative of 3 independent experiments.

Human cytokine array assays were performed to screen for cytokines that were differentially expressed between PC-3 prostate cancer cells and prostate cancer/monocyte-lineage co-cultures (Figure 2). The PC-3 High Invasive/U-937 co-culture supernatants showed increased CCL2 and Gro-α levels when compared to PC-3 High Invasive cancer cell supernatants alone (Figures 2A, 2B). In contrast, the co-culture supernatants showed high IL-6 and IL-8 levels similar to cancer cells alone. No significant differences were noted between cancer cell or co-culture supernatants in the remaining 38 cytokines or growth factors on the human cytokine array. Of note, granulocyte-colony stimulating factor (GCSF) and epidermal growth factor (EGF) were not increased in the co-culture supernatants (Figure 2B).

**Figure 3:**
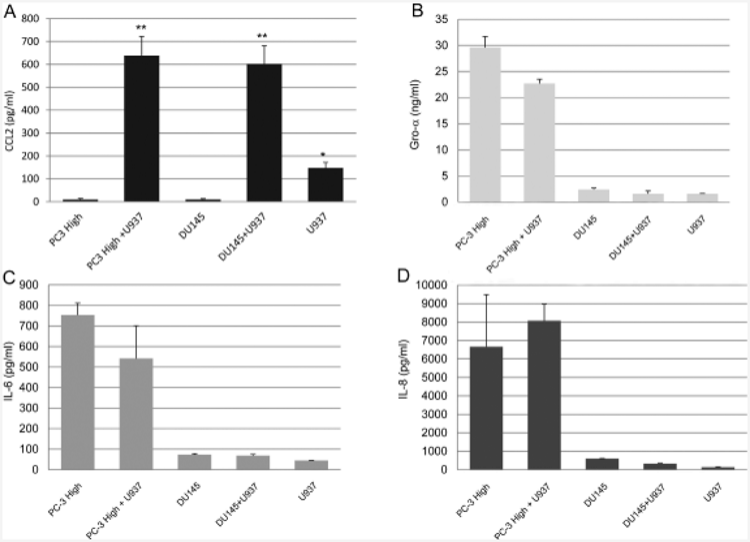
Selected cytokine levels of PC-3 High Invasion cells, DU145 and U-937 and co-culture supernatants. The supernatant cytokine levels were assayed by ELISA after 48 hours culture. (A) Co-cultures of U-937 cells with PC-3 High Invasion or DU145 cells yield increased CCL2 levels compared with cancer cell or U-937 supernatants alone. (B) High Gro-alpha levels were detected in PC-3 cell cultures and were decreased in the co-cultures and not quite statistically different, P=0.0561. (C). High Interleukin-6 levels were detected in PC-3 High Invasion cell cultures and were not significantly different in the co-cultures. (D) PC-3 High Invasion cell cultures yielded high Interleukin-8 levels which were not significantly different in the co-cultures. DU-145 yielded low Gro-alpha, IL-6 and IL-8 levels. The data are expressed as mean ± SD of four independent experiments. *P<0.01, **P<0.001.

**Figure 4:**
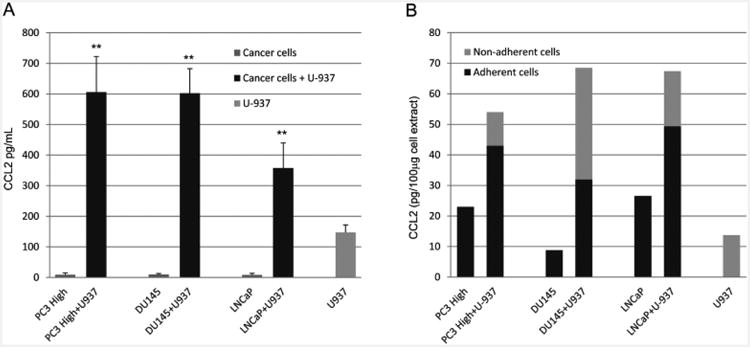
CCL2 levels from prostate cancer cells cultured alone or co-cultured with U-937 cells. (A) The PC-3 High Invasion prostate cancer cells cultured alone expressed very low supernatant levels of CCL2 compared with U-937 cells and PC-3 High Invasion/U-937 co-culture supernatants. Also, significantly increased CCL2 levels were detected in the supernatants of U-937 cells co-cultured with DU145 and LNCaP prostate cancer cells. Data are expressed as the mean ± SD of 4 independent experiments. **P<0.001. (B) Increased CCL2 levels (>30 pg/100 μg extract) were detected in the adherent cell extracts from co-cultures of PC-3 High Invasive, DU145 and LNCaP with U-937 cells when compared with the cancer cells alone. The non-adherent cell extracts from PC-3 High Invasive/U-937 and LNCaP/U-937 co-cultures showed similar CCL2 levels as the U-937 cell extracts. Non-adherent cell extracts from DU145/LNCaP co-cultures were increased above U-937 cell extracts. The data are representative of 4 independent experiments.

CCL2 was measured at very low levels (<10 pg/mL) by enzyme-linked immunosorbent assays (ELISA) in prostate cancer cell supernatants and U-937 supernatants (147.5 ± 24 pg/mL)(Figure 3A). In the PC-3 High Invasive/U-937 and DU145/U-937 co-culture supernatants, CCL2 increased to 638 ± 17 pg/mL and 600 ± 45 pg/mL, respectively (Figure 3A). Similarly, in LNCaP/U-937 co-culture supernatants, CCL2 was increased from less than 10 pg/mL to 358 pg/mL (Figure 4A).

The PC-3 High Invasive supernatants contained 29.6 ± 3.5 ng/mL Gro-α, compared with 22.7 ± 1.6 ng/mL in the co-culture supernatants (Figure 3B). The PC-3 supernatants contained 753 ± 165 pg/mL Interleukin 6, compared with 541 ± 315 pg/mL in the co-cultures (Figure 3C). The Gro-α and IL-6 levels were lower in the co-culture supernatants compared with the prostate cancer cells alone. The PC-3 High Invasive supernatants contained 6663 ± 2840 pg/mL Interleukin 8, similar to the co-culture supernatants with 8075 ± 925 pg/mL. In contrast, the DU145 and U-937 and co-culture supernatants showed low levels of Gro-α, IL-6 and IL-8 that were not increased in the co-cultures. Similarly, the LNCaP cell supernatants and co-cultures with U-937 cells expressed low levels of Gro-α, IL-6 and IL-8 (data not shown).

When co-cultured with U-937 cells, the PC-3 High Invasive cell extracts contained increased CCL2 (42 pg CCL2/100 μg) compared with the unstimulated PC-3 extracts (22 pg CCL2/100 μg) (Figure 4B). The DU145 and LNCaP cell extracts also showed increased CCL2 levels from co-cultures. The U-937 cell extracts and the co-cultured U-937 cell extracts contained similar low CCL2 levels (12 pg CCL2/100 μg) (Figure 4B). However, the U-937 cells co-cultured with DU145 cells exhibited increased CCL2 protein (36 pg CCL2/100 μg).

### Chemokine (C-C motif) ligand 2 (CCL2) stimulates PC-3 prostate cancer cell invasion

**Figure 5:**
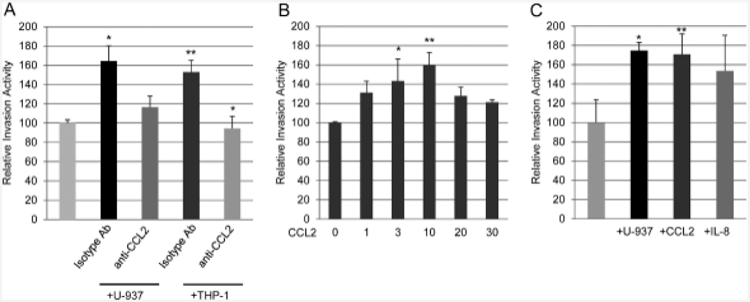
Effect of monocyte co-cultures and CCL2 on PC-3 High Invasive prostate cancer cell invasion. (A) PC-3 High Invasive cell invasion was induced by co-cultures with U-937 or THP-1 cells and was inhibited by anti-CCL2 neutralizing antibodies compared with isotype control antibodies. (B) PC-3 High Invasive cell invasion was induced by addition of purified recombinant human CCL2 protein to the lower Transwell chamber with optimum at 10 ng/mL CCL2. (C) PC-3 invasion activity was induced by addition of 10 ng/mL CCL2 or 5 ng/mL interleukin-8 to a similar degree as induced by U-937 co-cultures. The percent PC-3 cell invasion activity is expressed as mean ± SD of 3 independent experiments. *P<0.05; **P<0.001.

When PC-3 cells were co-cultured with U-937 or THP-1 monocytelineage cells their invasion activity was significantly increased above PC-3 control cells (Figure 5A). The co-cultured PC-3 cell invasion was inhibited towards control in the presence of anti-CCL2 neutralizing antibodies but not isotype controls (Figure 5A). Recombinant human CCL2 protein added to the lower well of the Transwell apparatus caused dose-dependent stimulation of PC-3 prostate cancer cell invasion (Figure 5B). The optimal PC-3 invasion occurred at 3 to 10 ng/mL added CCL2 protein. Interestingly, addition of high levels of CCL2 at 20 and 30 ng/mL did not stimulate prostate cancer cell invasion. Addition of recombinant CCL2 protein stimulated PC-3 invasion similar to the U-937 co-cultures (Figure 5C).

**Figure 6:**
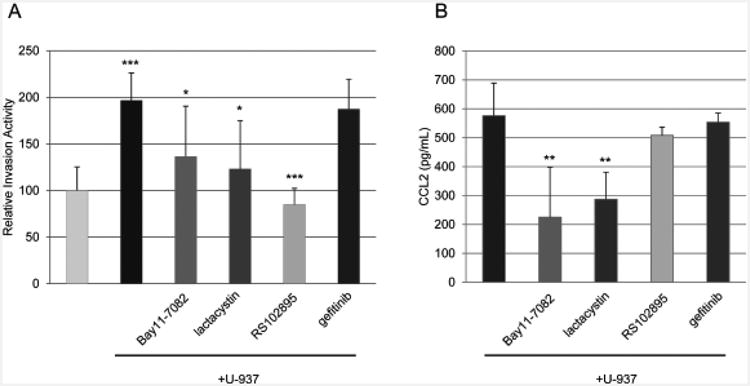
Effect of biochemical inhibitors on PC-3 High Invasive prostate cancer cell invasion activity and CCL2 levels in the co-culture with U-937 cells. (A) Prostate cancer cell invasion induced by co-culture with U-937 cells was inhibited with addition of Bay11-7082, Lactacystin and RS102895, but not with gefitinib. (B) CCL2 levels in co-culture supernatants were significantly reduced with Bay11-7082 or Lactacystin treatments, but not with RS102895 or gefitinib inhibitors. The data shown is expressed as the mean ± SD of three independent experiments. *P<0.05, **P<0.01, ***P<0.001, PC-3 High Invasive/U-937 versus with inhibitor or PC-3 High Invasive alone.

The role of CCL2 in U-937-induced PC-3 High Invasive cell invasion activity was tested with biochemical inhibitors (Figure 6A). Treatment with CCR2 inhibitor RS102895 reduced PC-3 invasion from 197 to 85 percent. Co-culture induced PC-3 invasion was also inhibited by treatment with Bay11-7082 (selective irreversible NF-κB inhibitor) and with Lactacystin (selective 20S proteosome inhibitor). The EGF signaling pathway was tested because EGF paracrine signaling was shown in some models to stimulate cancer cell invasion [7,8]. The EGFR inhibitor gefitinib did not inhibit PC-3 invasion in the co-cultures (Figure 6A). Bay11-7082 treatment inhibited CCL2 levels from 576 pg/mL to 224 pg/mL in the control co-cultures. Similarly, lactacystin inhibited CCL2 levels down to 287 pg/mL (Figure 6B). However, CCL2 levels in the co-culture supernatants were not significantly inhibited by treatment with RS102895 or gefitinib (Figure 6B).

### Co-cultures with U-937 cells and recombinant CCL2 induce PC-3 High Invasive prostate cancer cell NF-κB p65 DNA binding activity

**Figure 7:**
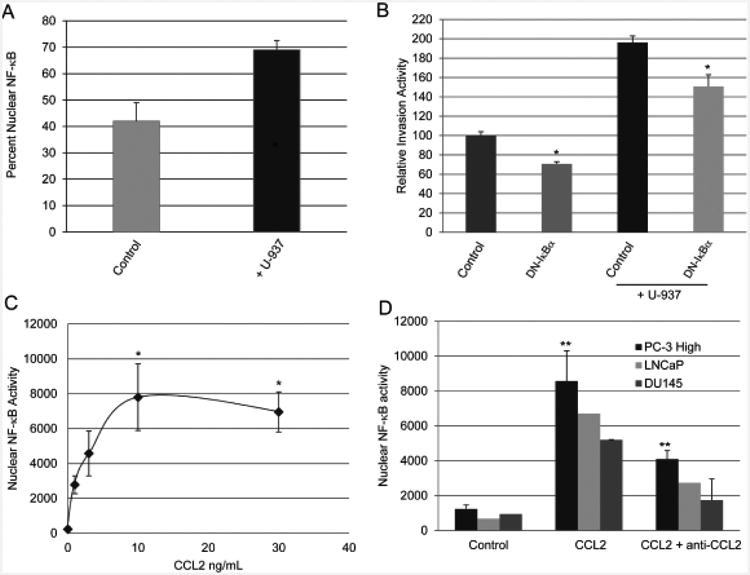
Prostate cancer cell invasion and NF-κB activity in co-cultures. (A) The percent nuclear NF-κB was significantly induced in PC-3 High Invasive prostate cancer cells co-cultured with U-937 monocytes versus alone. (B) Relative PC-3 invasion in co-culture with U-937 cells was reduced from 196 ± 7 to 151 ± 12 percent by transfection with dominant negative IκBαS32/36A versus control vector. (C) Dose dependent nuclear NF-κB DNA binding activity was induced in PC-3 High Invasive cells following incubation with recombinant human CCL2 for 24 hours. (D) The CCL2 induced PC-3 High Invasive cell NF-κB activity was reduced when the cells were treated with anti-CCL2 neutralizing antibodies. The data are expressed as the mean ± SD of three independent experiments. *P<0.05, **P<0.01.

PC-3 High Invasive prostate cancer cells co-cultured with U-937 cells showed increased nuclear NF-κB p65 compared with PC-3 cancer cells cultured alone (Figure7A). PC-3 transfection with IκBαS32/36A inhibited PC-3 invasion in the co-cultures when compared with control vector transfected PC-3 cells (Figure 7B). Transfection of the PC-3 cells with dominant negative IκBα S32/36A expression vector inhibited NF-κB-Luciferase reporter and NF-κB DNA binding activity compared with controls (Supplementary Figure 1).

Treatment of PC-3 High Invasive prostate cancer cells with recombinant human CCL2 protein induced dose-dependent nuclear NF-κB p65 DNA binding activity (Figure 7C). Addition of 10 ng/mL CCL2 caused optimal induction of PC-3 cell NF-κB DNA binding activity (Figure 7C). Treatment with 30 ng/CCL2 did not further increase PC-3 NF-κB activity. In contrast, anti-CCL2 neutralizing antibodies inhibited nuclear NF-κB DNA binding activity in PC-3, DU145 and LNCaP cells treated with 10 ng/mL recombinant CCL2 (Figure 7D). These experiments taken together show that the co-cultures and recombinant CCL2 protein similarly stimulated prostate cancer cell invasion and NF-κB activity.

### Prostate cancer tissues contain tumor-associated macrophages and express epithelial CCL2 and increased nuclear NF-κB subunits

**Figure 8:**
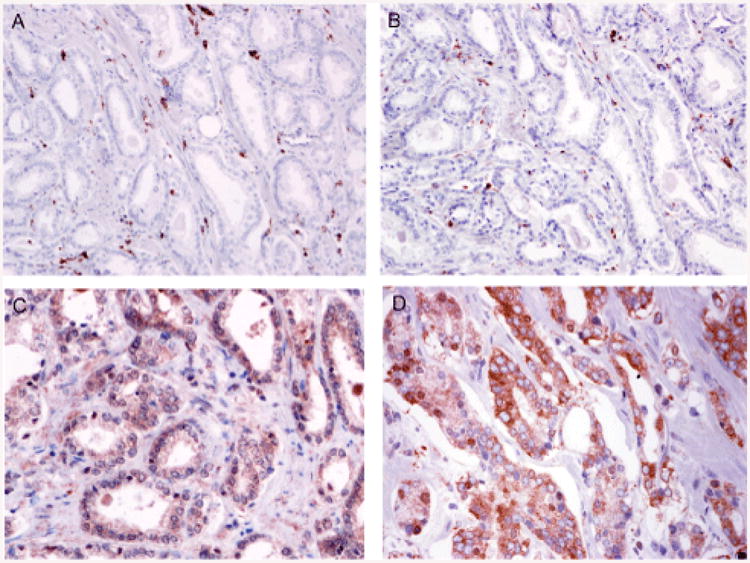
Prostate immunohistochemistry with tumor associated macrophages. (A) CD68 positive cells are demonstrated in the stroma adjacent to the prostate cancer epithelium (20×). (B) CD206 (mannose receptor) positive cells are shown in the stroma adjacent to the prostate cancer epithelium (20×). (C) Prostate cancer immunostained with anti-CCL2 (CCL2) showing epithelial and stromal cell expression (40×). (D) Prostate cancer immunostained showing nuclear and cytoplasmic NF-κB p65 (40×).

**Figure 9:**
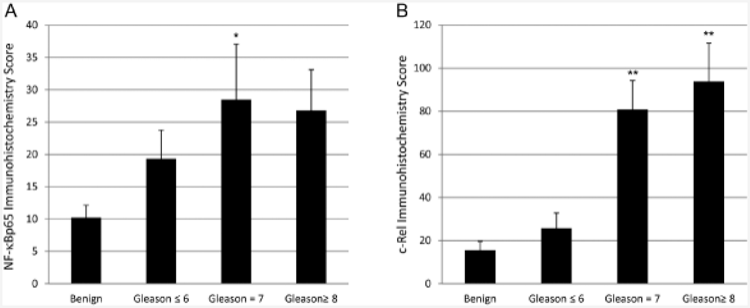
Comparison of NF-κB and c-Rel immunostaining scores from clinical prostate tissue samples. The scores represent the product of percent positive nuclei times the immunostain scoring intensity. The data are expressed as the mean ± SEM of 15 benign and 13 prostate cancer tissue samples, *P<0.002, **P<0.0001.

Prostate cancer tissues were immunostained to analyze prostate cancer macrophage, CCL2 and NF-κB subunit localization (Figure 8A-8D). Prostate sections contained CD68 and CD206 (macrophage mannose receptor) positive cells in the stroma (Figures 8A, 8B). The prostate cancer epithelium contained CCL2 positive cells (Figure 8C). NF-κBp65 was observed within the cytoplasm and nucleus of the prostate cancer epithelium (Figure 8D). The fraction of NF-κB positive cells ranged up to 70 percent. A representative section shows NF-κBp65 positive staining in 10 percent of the cancer cells (Figure 8D). The NF-κBp65 and c-Rel immunohistochemistry scores in cancer containing prostate tissues correlated with Gleason's score and were increased compared with biopsy samples containing benign epithelium (Figure 9).

## Discussion

When prostate cancer invades and spreads outside of the prostate, the disease becomes very difficult to control and cure. Inflammatory cells within the tumor microenvironment are now thought to play an important role in cancer development and progression. In the present study, we investigated interactions between prostate cancer and monocyte-lineage cell lines to learn how this process can contribute to prostate cancer invasion and progression. The prostate cancer cells showed increased invasion without increased proliferation when co-cultured with U-937 or THP-1 monocyte-lineage cells (Figure 1) [40].

In this study, a cytokine antibody array was used to screen for differential expression of 42 different cytokines and growth factors in the cancer cell and co-culture supernatants. Four of the cytokines screened in this array were present at high levels in the co-culture supernants; however, only the MCP-1/CCL2 cytokine was increased in the co-cultures above the cancer cells alone when tested by ELISA. MCP-1/CCL2 expression was increased 4-fold in the co-cultures above monocytes and more than 40-fold above cancer cells cultured alone. The CCL2 expression increased in both the prostate cancer cells and monocytes with the greatest increase in the cancer cells. Although MCP-1/CCL2 was produced by both monocytes and prostate cancer cells, it appears that the CCL2 production was not simply driven by a positive feedback loop, since the CCR2 inhibitor RS102895 did not affect the CCL2 levels in the co-cultures. However, there may be other cytokines or factors that may not have been detected in this cytokine array screen, including bioactive lipids that could have stimulated the cancer cells.

Recombinant CCL2 protein stimulated prostate cancer cell invasion in a dose-dependent manner, similar to the co-cultures with monocyte-lineage cells. Previous studies have shown that RhoA and NF-κB activity were essential for high PC-3 prostate cancer cell invasion [31,32]. The current study demonstrates that prostate cancer/monocyte co-cultures expressed high levels of CCL2 which can stimulate prostate cancer cell NF-κB activity and invasion. This study demonstrated that PC-3 prostate cancer cell NF-κB activity was also stimulated by the co-cultures and CCL2 treatment. These effects were largely blocked by introducing NF-κB inhibitors to the prostate cancer cells. These data demonstrate for the first time cross-communication between prostate cancer cells and monocytes involving increased CCL2 expression with increased prostate cancer cell NF-κB activity and invasion.

CCL2 is produced by stromal cells, osteoblasts, endothelial and cancer cells in the microenvironment and can stimulate monocyte recruitment, osteoclast maturation and cancer cell growth and survival [21]. CCL2 is expressed in clinical prostate cancer tissues and its expression has been correlated with Gleason's score and pathological stage [15,22,41]. Prostate cancer cells also express the primary CCL2 receptor, CCR2, which was increased on prostate cancer cells during clinical progression [22,42,43]. In the present study, the prostate cancer cells and co-cultures also expressed high levels of IL-6, IL-8 and Gro-α; however, only CCL2 levels increased significantly in the co-cultures with monocytes. It is not known why Gro-alpha and IL-6 levels were lower in the co-cultured cells by ELISA when the cytokine array showed higher levels of these factors. It is possible that the cytokine ELISA did not confirm the increased expression of Gro-alpha and IL-6 in the co-cultures due to analytical variation in the assays.

The mechanisms by which CCL2 may stimulate prostate cancer cell invasion are not known, but CCL2 may increase cancer cell adhesion, migration and proteolysis [22,23]. Newly demonstrated, prostate cancer cells were stimulated to increased invasion by CCL2 and monocyte co-cultures in a NF-κB-dependent manner. The data in this study showed that increased prostate cancer cell invasion and NF-κB activation were induced by high CCL2 expression found in the co-cultures. Previously, very limited evidence suggested that CCL2 may stimulate NF-κB signaling in cancer cells [44]. The increased prostate cancer cell nuclear c-Rel and NF-κB p65 expression in clinically advanced prostate cancer tissues where increased macrophage counts and CCL2 expression have been observed further supports the role of NF-κB activity in prostate cancer progression [15,40,45].

Increased NF-κB activity has also been reported in head and neck, lung, breast, pancreas and colorectal cancers [3,27,46]. NF-κB is a key transcriptional regulator of pro-inflammatory cytokines and extracellular proteases that promote cancer cell survival, adhesion and invasion [47,48]. In models of inflammation-associated cancer, cytokine and NF-κB signaling contributed to cancer development through myeloid growth factor expression, cancer cell epithelial-to-mesenchymal transition and cell survival [26,27,49-51]. Further, stromal inflammatory cytokines have been shown to activate cancer cell NF-κB activity and tumor progression [52-54]. NF-κB is a critical factor for epithelial-to-mesenchymal transition (EMT) processes important to cancer dissemination [51,55]. Previous studies in our group have shown that NF-κB is an essential downstream mediator of TGF-β-induced prostate cancer cell vimentin expression and EMT [51]. NF-κB and vimentin expression also correlated with increasing pathological grade and biochemical recurrence of clinical prostate cancer. NF-κB activity has also been shown to be essential for activation of cytokine and extracellular protease expression necessary for prostate cancer invasion and metastasis [30,34,35]. However, to our knowledge, this report first demonstrates that CCL2 in monocyte co-cultures activated prostate cancer cell invasion through increased NF-κB activity [44,56-58].

Tumor specific paracrine stimulation between cancer cells and immune cells has been observed. In human breast and ovarian cancers, immune cells induced cancer cell invasion by TNF-α stimulation and increased expression of factors influencing invasion [25,59]. In a murine mammary carcinoma model, CSF-1 produced by tumor cells stimulated tumor macrophages to produce epidermal growth factor (EGF) which stimulated cancer cell chemotaxis and microvessel intravasation [7,60]. In the present study, the cytokine array revealed increased MCP-1/CCL2 but not GCSF or EGF expression in the co-cultures. The experiments also demonstrated that CCL2 was the key factor increased in the co-cultures that stimulated prostate cancer cell invasion and NF-κB activity. These examples suggest that tumor specific molecular targets may regulate tumor growth and progression.

## Conclusions

This study demonstrated an interaction between prostate cancer cells and monocyte-lineage cells causing increased prostate cancer invasion. In this model system, cross-communication significantly increased CCL2 expression by both prostate cancer and U-937 cells and lead to increased prostate cancer cell invasion and NF-κB activation. The increased prostate cancer cell invasion depended on high CCL2 levels and was significantly inhibited by CCL2 neutralizing antibodies or treatment with a biochemical CCR2 inhibitor. Moreover, prostate cancer cell invasion stimulated by monocytes or recombinant CCL2 depended on prostate cancer cell NF-κB activity. These findings suggest that CCL2 and NF-κB may be critical mediators of monocyte-induced prostate cancer cell invasion and may serve as therapeutic targets to interfere with inflammation-associated prostate cancer progression.

## Supplementary Material

Supplementary Figure 1

Supplementary Figure legend

Supplementary file
