# Supplementary figures and images for: Monocyte-Induced Prostate Cancer Cell Invasion is Mediated by Chemokine ligand 2 and Nuclear Factor-κB Activity
Source: J Clin Cell Immunol. Author manuscript; Available in PMC 2015 Aug 25. (PMC4548876; doi:10.4172/2155-9899.1000308)

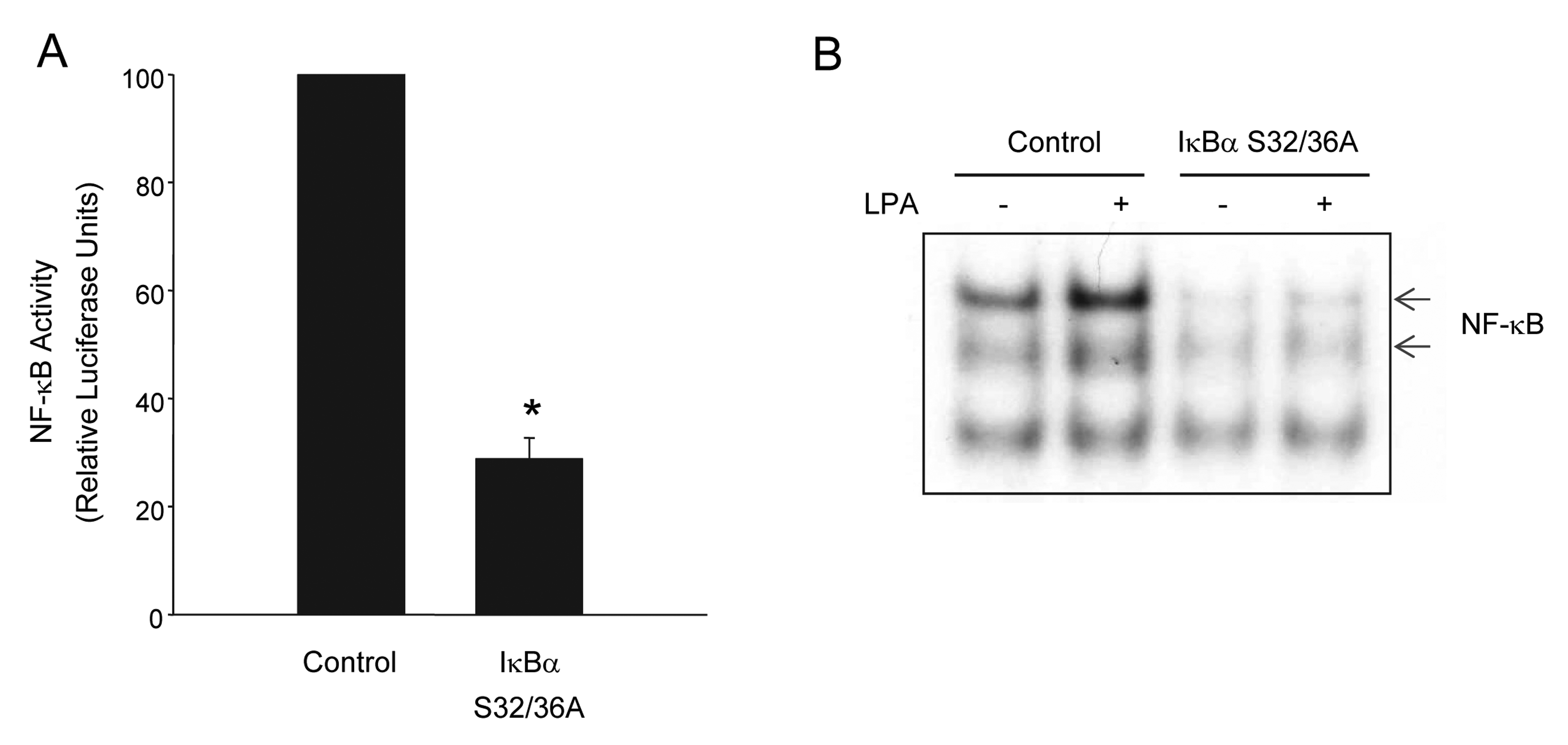

Supplement: Supplementary Figure 1 [file NIHMS699109-supplement-Supplementary_Figure_1.tif]
