# Supplementary material for: Monocyte-Induced Prostate Cancer Cell Invasion is Mediated by Chemokine ligand 2 and Nuclear Factor-κB Activity
Source: J Clin Cell Immunol. Author manuscript; Available in PMC 2015 Aug 25. (PMC4548876; doi:10.4172/2155-9899.1000308)
Supplement: Supplementary Figure legend [file NIHMS699109-supplement-Supplementary_Figure_legend.docx]

Supplementary Figure 1. (A) NF-κB activity of PC-3 High Invasive cells transfected with pCMV 4-3 HA IκB*α* S32/36A or pCMV 4-3 HA control vector. The relative luciferase activity was measured in PC-3 High Invasive cells co-transfected with pNF-κB-Luc or control vector. The NF-κB activity as relative luciferase units percent of control is expressed as mean ± SEM of 9 experiments. (B) Expression of dominant negative IκBα S32/36A blocked constitutive and lysophophatidic acid-induced NF-κB DNA binding activity in the gel shift assay (compare lanes 1 and 3 and lanes 2 and 4, respectively).
