# Supplementary material for: Monocyte-Induced Prostate Cancer Cell Invasion is Mediated by Chemokine ligand 2 and Nuclear Factor-κB Activity
Source: J Clin Cell Immunol. Author manuscript; Available in PMC 2015 Aug 25. (PMC4548876; doi:10.4172/2155-9899.1000308)
Supplement: Supplementary file [file NIHMS699109-supplement-Supplementary_file.zip › Monocytes and PCa cancer cell invasion.pdf]

Role of Monocyte-Lineage Cells in Prostate Cancer Cell Invasion and Tissue Factor Expression

Paul F. Lindholm,[1]* Yi Lu,[3] Brian P. Adley,[1] Tudor Vladislav,[3] Borko Jovanovic,[4] Neela Sivapurapu,[1] Ximing J. Yang,[1,2] and André Kajdacsy-Balla[3]

[1]Department of Pathology, The Feinberg School of Medicine, Northwestern University, Chicago, Illinois
[2]Robert H. Lurie Cancer Center, Prostate Cancer Tissue Core Facility, The Feinberg School of Medicine, Northwestern University, Chicago, Illinois
[3]Department of Pathology, College of Medicine, University of Illinois, Chicago, Illinois
[4]Department of Preventive Medicine and Bioinformatics Core, The Feinberg School of Medicine, Northwestern University, Chicago, Illinois

BACKGROUND. Tissue factor (TF) is a cell surface glycoprotein intricately related to blood coagulation and inflammation. This study was performed to investigate the role of monocyte-lineage cells in prostate cancer cell TF expression and cell invasion.
METHODS. Prostate cancer cell invasion was tested with and without added peripheral blood monocytes or human monocyte-lineage cell lines. TF neutralizing antibodies were used to determine the TF requirement for prostate cancer cell invasion activity. Immunohistochemistry was performed to identify prostate tissue CD68 positive monocyte-derived cells and prostate epithelial TF expression.
RESULTS. Co-culture of PC-3, DU145, and LNCaP cells with isolated human monocytes significantly stimulated prostate cancer cell invasion activity. TF expression was greater in highly invasive prostate cancer cells and was induced in PC-3, DU145, and LNCaP cells by co-culture with U-937 cells, but not with THP-1 cells. TF neutralizing antibodies inhibited PC-3 cell invasion in co-cultures with monocyte-lineage U-937 or THP-1 cells. Prostate cancer tissues contained more CD68 positive cells in the stroma and epithelium ($145 \pm 53/mm^2$) than benign prostate ($108 \pm 31/mm^2$). Samples from advanced stage prostate cancer tended to contain more CD68 positive cells when compared with lower stage lesions. Prostatic adenocarcinoma demonstrated significantly increased TF expression compared with benign prostatic epithelium.
CONCLUSIONS. This study shows that co-culture with monocyte-lineage cells induced prostate cancer cell invasion activity. PC-3 invasion and TF expression was induced in co-culture with U-937 cells and partially inhibited with TF neutralizing antibodies. *Prostate*
© 2010 Wiley-Liss, Inc.

KEY WORDS: **prostate cancer; monocytes; tissue factor; invasion**

INTRODUCTION

Inflammation and infection contributes to the development of several gastrointestinal, head and neck and uterine cervical cancers and other cancer types [1–8]. The association of chronic inflammation and infection with prostate cancer has also been studied and a history of previous venereal infections has been epidemiologically linked to an increased risk of prostate cancer development [9]. In addition, areas of prostate inflammation have been found to occur in proximity with proliferating prostate epithelium, or

Grant sponsor: Department of Defense New Investigator; Grant number: DAMD17-02-1-0162; Grant sponsor: National Cancer Institute Prostate; Grant number: P50-CA090386.
Current address: Brian P. Adley, MD, Advocate Lutheran General Hospital, Park Ridge, IL.
*Correspondence to: Paul F. Lindholm, Department of Pathology, The Feinberg School of Medicine, Northwestern University, 303 East Chicago Avenue, Chicago, IL 60611.
E-mail: p-lindholm@northwestern.edu
Received 9 October 2010; Accepted 3 May 2010
DOI 10.1002/pros.21202
Published online in Wiley InterScience (www.interscience.wiley.com).

© 2010 Wiley-Liss, Inc.

postinflammatory atrophy and developing prostate cancer lesions [10–12].

The tumor microenvironment contains stromal cells and host inflammatory cells including tumor-associated macrophages (TAMs), which likely interact with and promote aggressive cancer cell behavior [4,6,13–16]. In head and neck squamous cancers, increased tumor-associated macrophage cell content has been associated with increased tumor size and extracapsular extension, and lymph node metastatic spread [17,18]. TAMs have been found in clinically aggressive tumors including prostate cancer but their role is presently incompletely understood [19,20]. Inflammatory cells and TAMs produce proinflammatory cytokines and chemokines including macrophage inhibitory cytokine-1 (MIP-1); monocyte chemoattractant protein-1 (MCP-1); IL-8 and reactive oxygen, nitric oxide, and bioactive lipids that may promote tumor cell proliferation and migration [1,6,13,21,22]. The cross communication of tumor cells and inflammatory cells may lead to increased expression of additional factors in cancer cells including Cox-2, MMPs, β1 integrin, and tissue factor (TF) that may promote their survival, adhesion, invasion, and metastasis [6,23,24]. In experimental studies, inflammatory cells stimulated tumor growth through NF-κB activity and inflammatory mediators [25,26]. TF may be one important NF-κB regulated factor that could promote aggressive cancer cell behavior [27–32].

TF is a 47 kDa transmembrane glycoprotein receptor that binds to coagulation factor VIIa to activate the coagulation cascade while transmitting intracellular signals affecting cellular growth and motility [33]. TF levels are increased in several aggressive and high-grade cancers including malignant melanoma, non-small cell lung cancer (NSCLC), pancreatic, colorectal, and prostate carcinoma [29,30,34–38]. While TF is highly expressed in several cancer types and may promote tumor progression, less is known about factors that lead to TF upregulation in cancer cells and precisely how it may promote tumor progression [39–42].

This study was performed to test the interaction between cells of monocyte-lineage and epithelial TF expression in prostate cancer. The in vitro co-culture of monocyte-lineage cells induced higher invasion activity in prostate cancer cells, which was partially blocked by TF neutralizing antibodies. Further, histological studies showed a correlation between stromal CD68 positive cells and prostate cancer epithelial TF expression.

MATERIALS AND METHODS

Cell Culture

Human prostate cancer cells PC-3, LNCaP, DU145, and monocytoid U-937 and THP-1 cell lines were purchased from ATCC (Rockville, MD). The PC-3 high- and low-invasive cell lines were selected by three serial passages through Matrigel® reconstituted basement membranes (Becton Dickinson, Lincoln Park, NJ) in a Transwell® chamber with 8 μM pore size [43]. These selected cell populations are referred to as PC-3 highly invasive and PC-3 low-invasive cells in previous publications [43,44]. The PC-3 EGFP and RhoA Q63E stable cell lines were created by LipofectAMINE-mediated transfection of the PC-3 highly invasive cells with a constitutively active RhoA Q63E-EGFP-C1 or control vector pEGFP-C1 (Clontech, Palo Alto, CA) followed by continual selection with 1.5 mg/ml G418 (Life Technologies, Inc.) [44]. All cells were maintained in a humidified atmosphere of 5% CO_2 at 37°C in RPMI 1640 medium supplemented with 10% fetal bovine serum (FBS; Biofluids, Rockville, MD); 2 mM L-glutamine; 100 units/ml penicillin, and 100 μg/ml streptomycin (Life Technologies, Inc.).

Transwell Invasion Assay

The invasion assay was performed by adding 50,000 [^{3}H] Thymidine (GE Healthcare Bio-Sciences, Piscataway, NJ) pulse-labeled cells to the upper chamber coated with 35 μg Matrigel (Becton Dickinson) separated from the lower chamber by 8 μm pores in Transwell chamber plates (Costar, Corning, NY) [44]. The effect of human monocytes on prostate cancer cell invasion was tested using Matrigel-coated Transwell chambers as previously described [43–46]. The cancer cell invasion assay was cultured with or without 10,000 U-937 or THP-1 monocytoid cell lines (ATCC) or 20,000 human peripheral blood monocytes added to the lower chamber. The human monocytes were isolated from normal donor peripheral blood by mononuclear cell adherence to plastic dishes [47]. The cells were cultured in RPMI 1640 medium supplemented with 10% FBS [43,45]. The cell invasion assays were tested in triplicate and each experiment was performed three or more times. In this assay, the labeled cancer cells that passed through the Matrigel membrane into the lower Transwell chamber were counted and compared to the total number of labeled cells. The percentage of invaded cells in the lower chamber at 72 hr was determined by multiplying by 100, the cell associated ^{3}H-thymidine cpm (recovered from each lower chamber by trypsinization) divided by the total cell-associated cpm initially added to each chamber. The percent invasion was not affected by cell proliferation or viability as determined by viable cell counts [44].

TF neutralizing monoclonal antibodies 5B7 and 10H10 were obtained from Dr. Gerald Soff and Dr. James Morrissey. For TF neutralizing experiments, the cultures were incubated with anti-TF neutralizing

antibodies ranging from 0.25 to 2.0 μg/ml. Reproducible, dose-dependant inhibition of PC-3 invasion was found with optimal inhibition at 1.0 and 2.0 μg/ml of neutralizing antibodies (data not shown). For subsequent experiments, TF neutralizing antibodies were used at a 1 μg/ml concentration and isotype control monoclonal antibodies were also used at 1 μg/ml.

ELISA

The TF (Thromboplastin, factor III) protein expression of prostate cancer cell extracts was measured with the Human TF ELISA Kit (AssayPro, St. Charles, MO). Extracts were made from the cancer cells grown to 70–80% confluence by incubating with cell extraction buffer containing protease inhibitor cocktail at 4°C. The microcentrifuge cleared cell extracts were tested in duplicate and incubated with precoated micro-test strips overnight at 4°C. The assay conditions were performed per kit instructions. The absorbances were determined on a micro-test plate reader at a wavelength of 450 nm within 30 min. The TF was quantified for each sample from a standard curve made with TF standards from 0 to 400 pg/ml. The TF measured for the cell extract are expressed as picogram per microgram (pg/μg) of cell extract applied to the assay.

Cell Proliferation and Viability Assays

The Vybrant™ MTT Cell Proliferation Assay Kit (Molecular Probes, Eugene, OR) was used to measure cell proliferation as previously described [44,46]. Parallel determinations of cell number and viability were made by counting cells on a hemocytometer slide using the Trypan Blue exclusion technique.

Patient Samples and Tissue Microarray Construction

Tissue Microarrays were prepared from clinical prostatectomy specimens by the Robert H. Lurie Clinical Cancer Center Prostate S.P.O.R.E. Tissue Core facility. The prostatectomy specimens were obtained from patients with prostate cancer, which contained areas of benign prostate epithelium ($N = 24$) and cancer ($N = 23$) including low- (Gleason's score ≤ 6), intermediate- (Gleason's score $= 7$), and high-grade carcinoma (Gleason's score ≥ 8), and lymph node metastases for the tissue microarray. The tissue microarrays were assembled with 1.5 mm diameter cores representing low-, intermediate-, and high-grade prostate cancer as well as benign epithelium. Each prostatectomy specimen was represented by 3–5 formalin-fixed, paraffin-embedded tissue cores on the tissue microarray.

Immunohistochemistry

For this study, the prostate tissues were immunostained with antibodies to CD68 (Dako clone; PG-M1) and TF (#4509, American Diagnostica). Each primary antibody was tested for optimal reactivity with serial dilutions following antigen retrieval. The stromal CD68 positive cells were quantified in five high power fields from immunostained tissue sections corresponding to the tissue microarray samples and the average determined for each prostate tissue sample. The non-luminal prostate tissue area was determined by masking glandular lumina followed by counting the number of total pixels for each histopathological prostate type using the Aperio ScanScope CS Positive Pixel Count algorithm (Aperio, Vista, CA). To correct for the differing tissue areas for each prostate histopathology type, the CD68 counts were normalized for mm^2 tissue area (stroma and epithelium).

The TF immunoreactivity was quantified by digital scoring using ChromaVision Automatic Cellular Imaging System II (ACIS II) [48] and average manual scoring independently by two general pathologists (Y.L. and P.F.L.), and a genitourinary pathologist (B.A.). The manual scoring was performed independently and without knowledge of the digital scoring. For pathologist manual scoring, the immunostaining intensity was graded using scoring criteria: 0, negative; 1+, weak positive; 2+, intermediate positive; and 3+, strong positive [49,50]. The digital image score was derived from intensity scores and percent of positive immunostaining areas. An ACIS II assisted Region Score was generated from the 0 to 255 intensity score and the percentage brown staining area in the selected region using the ACIS II software.

Statistical Analysis

Results are expressed with mean ± standard deviation. Statistical analysis was performed using GraphPad Prism version 3.00 for Windows, GraphPad Software (San Diego, CA, "www.graphpad.com"). The non-parametric Mann–Whitney test was used to compare prostate CD68 positive cell counts and prostate cancer biomarker expression between two groups. Where appropriate, Student's *t*-test was used for mean comparisons of the invasion and proliferation assays. Differences are considered significant when $P < 0.05$.

RESULTS

Effect of Human Monocyte Co-Cultures on Prostate Cancer Cell Basement Membrane Invasion

The PC-3 highly invasive cells cultured alone demonstrated 7.6 ± 1.0% invasion and when human monocytes were added to the invasion assay, the PC-3 cell invasion increased to 15.5 ± 3.4% (Fig. 1). Similarly, the invasion of DU145 cells without added monocytes

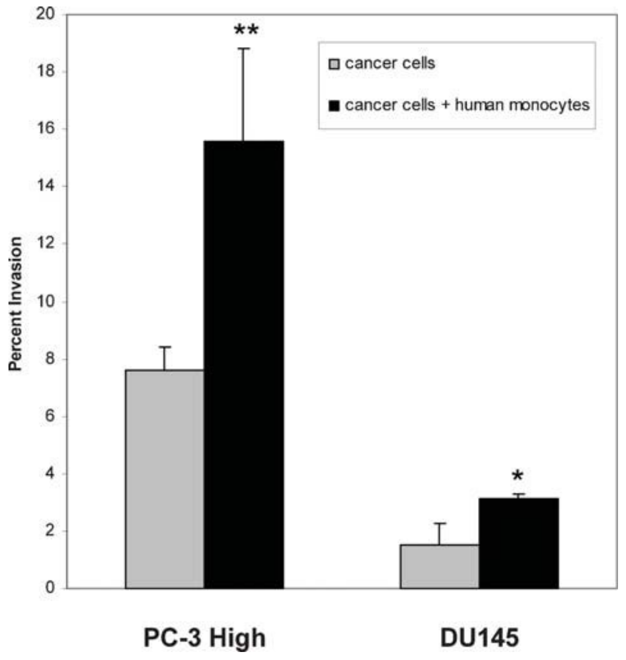

Fig. 1. Effect of human monocyte co-culture on prostate cancer invasion activity. Human monocytes isolated from normal human donors were placed in the lower chamber of a Transwell® apparatus. Co-culture of the prostate cancer cells with the human monocytes lead to significantly increased prostate cancer cell invasion when compared to the cancer cells cultured alone. Data expressed as mean ± SD; **P* < 0.05; ***P* < 0.001.

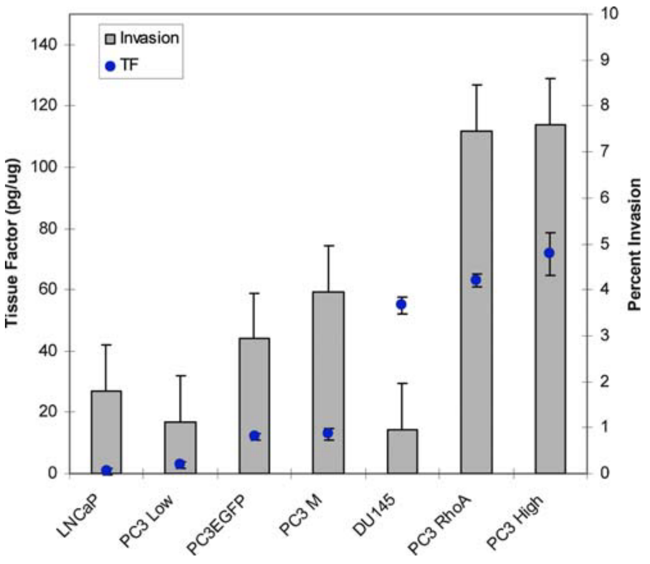

Fig. 2. Association between prostate cancer cell tissue factor (TF) expression and invasion activity. Selected human prostate cancer cell sub-lines were tested for invasion activity in the Matrigel-coated Transwell invasion chambers and cell extracts were tested for TF protein (picograms per microgram) with whole cell extracts using a TF ELISA.

was 1.4 ± 0.9% and with added human monocytes DU145 invasion increased to 3.0 ± 0.3%. The co-culture of the cancer cells with human monocytes did not change cell proliferation or viable cell number under similar conditions [44]. Thus, the co-culture with human monocyte-like cells led to a greater than twofold increase in PC-3 and DU145 prostate cancer cell invasion.

Effect of Monocyte-Lineage Cell Co-Cultures on Prostate Cancer Cell Basement Membrane Invasion and Tissue Factor Expression

The relationship between prostate cancer cell invasion and TF expression is shown for several prostate cancer cell lines (Fig. 2). Low-TF protein expression (<20 pg/μg) was observed in LNCaP, PC-3 low invasion, PC-3-EGFP, and PC-3M cell lines which also demonstrated low-invasion activity. In contrast, high-TF expression levels (greater than 60 pg/μg) were found in PC-3-RhoA and PC-3 highly invasive cell sub-lines. DU145 was the only cell line tested with low-invasion activity that showed high-TF expression. Overall, there was a positive relationship between TF expression and invasion activity in several of the prostate cancer cell lines. Co-culture of human monocyte-derived U-937 cells with prostate cancer cell lines stimulated TF expression 2.1-fold in LNCaP cells; 1.7-fold in DU145 cells and 1.4-fold for PC-3 cells (Fig. 3). In contrast, co-culture of THP-1 cells with these prostate cancer cell lines did not significantly increase prostate cancer cell TF expression.

Blocking experiments were performed with TF neutralizing and isotype control antibodies (see Materials and Methods Section) to determine the requirement of TF expression for PC-3 high-invasion activity (Fig. 4A). PC-3 highly invasive cells demonstrated 7.7 ± 1.1% invasion when incubated with non-immune isotype-control monoclonal antibodies compared with 5.5 ± 1.4% when the co-cultures were incubated with anti TF antibodies. By comparison, the PC-3 low-invasion selected cells showed 1.1 ± 0.3% invasion under the same conditions. Co-culture of the PC-3 highly invasive cells with human U-937 cells stimulated PC-3 invasion to 12.5 ± 1.8% (Fig. 4B). THP-1 cells stimulated PC-3 high-invasion activity to 13.1 ± 1.4% (Fig. 4C). When TF neutralizing antibodies were added to the PC-3 cells co-cultured with U-937 or THP-1 cells, their invasion was significantly inhibited to 8.6% and 8.5%, respectively.

CD68 Positive Cell Content of Benign Prostate and Prostate Cancer Tissue

The prostate content of monocyte-lineage cells was measured by CD68 immunohistochemistry. CD68

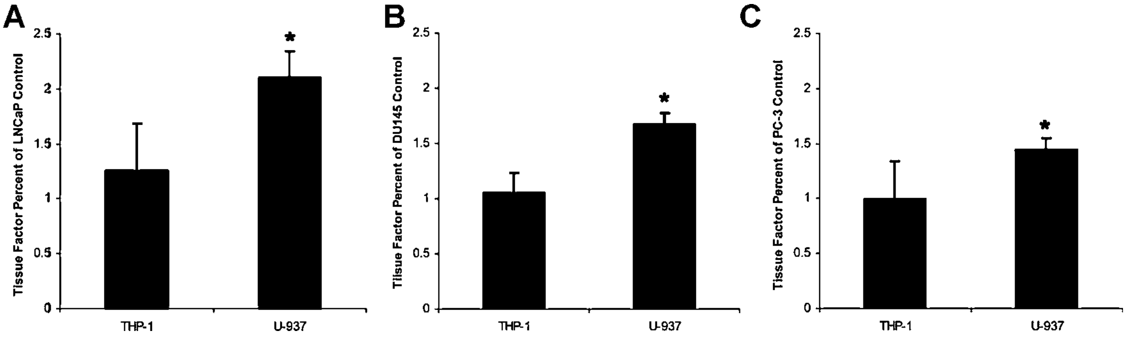

Fig. 3. The effect of monocyte-derived cell co-cultures on prostate cancer cell tissue factor (TF) expression. Co-cultures with U-937 cells, but not THP-1 cells significantly stimulated increased TF expression in (**A**) LNCaP cells; (**B**) DU145 cells; and (**C**) PC-3 cells. Data expressed as mean percent of control invasion ± SD; *$P < 0.05$.

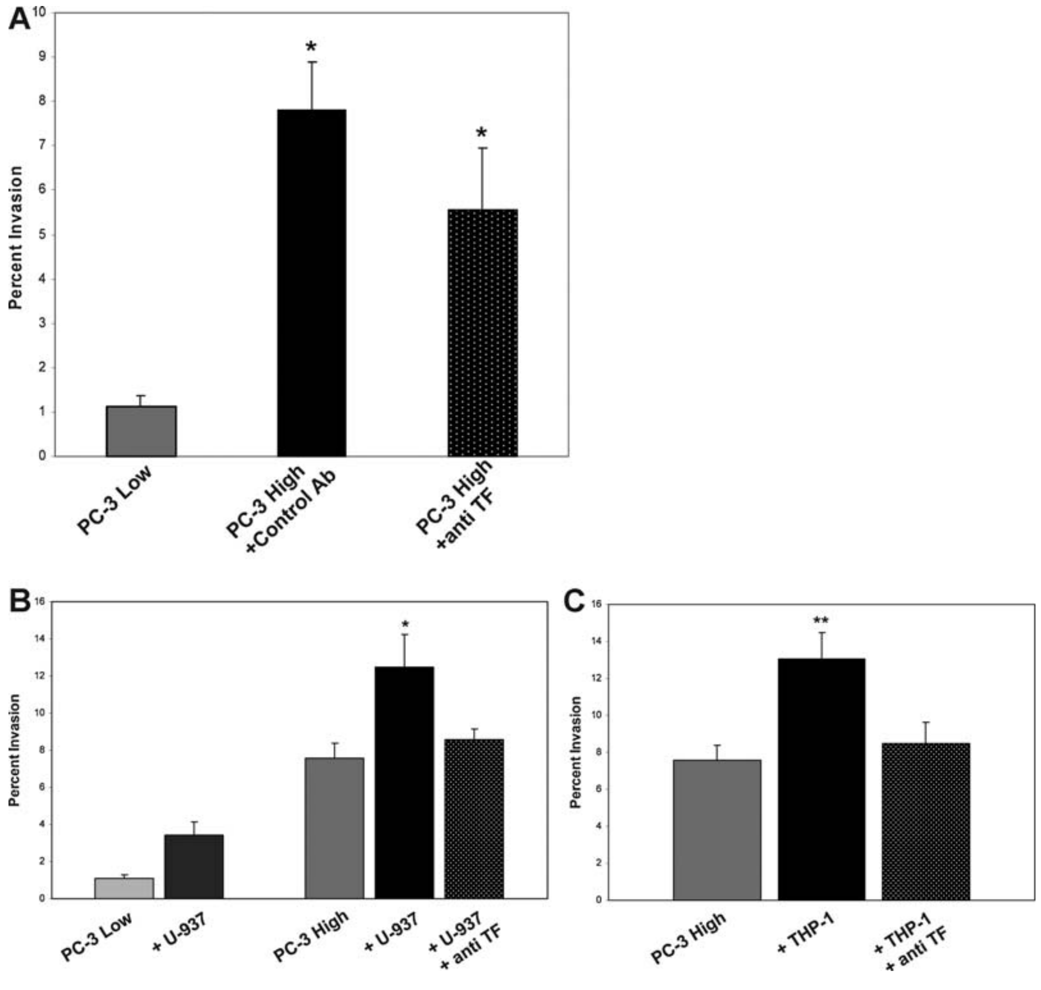

Fig. 4. Effect of tissue factor (TF) neutralizing antibodies on prostate cancer cell basement membrane invasion in monocyte co-cultures. **A**: The PC-3 high-invasion prostate cancer cell invasion activity was significantly reduced when anti-TF antibodies were added compared with control antibodies (Control Ab), expressed as mean ± SD; *$P < 0.001$. **B**: Co-culture of prostate cancer cells with the U-937 cell line leads to significantly increased invasion of PC-3 low- and PC-3 high-invasion cells when compared to the cancer cells cultured alone. TF neutralizing antibodies reduced U-937-induced PC-3 high-invasion cells to levels comparable to PC-3 cells alone; *$P < 0.001$. **C**: Co-culture of prostate cancer cells with the THP-1 cells lead to significantly increased PC-3 high-invasion cells when compared to the PC-3 high-invasion cells cultured alone. TF neutralizing antibodies reduced THP-1-induced PC-3 invasion to levels comparable to PC-3 cells alone; **$P < 0.01$. The data shown are representative of three independent experiments.

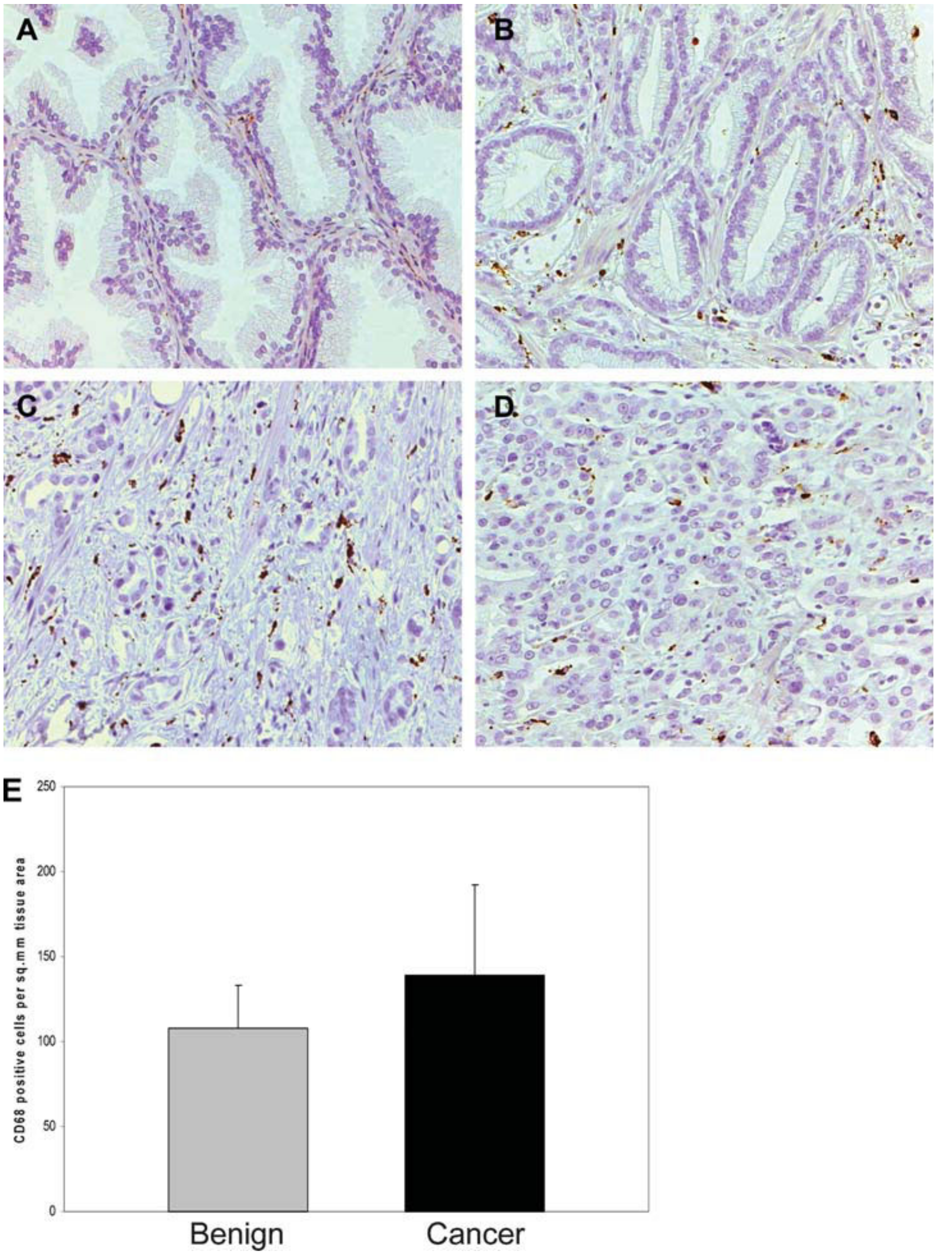

Fig. 5. Prostate immunohistochemistry and quantification of CD68 positive cells. **A**: CD68 positive cells in benign prostate stroma. **B**: CD68 positive cells in low-grade cancer stroma. **C**: CD68 positive cells in high-grade prostate cancer. **D**: CD68 positive cells in a prostate cancer lymph node metastasis. **E** Quantification of CD68 positive cells per mm^2 tissue area of benign and prostate cancer tissue (excluding gland lumina) is expressed as the mean $\pm$ SD; $^*P = 0.063$.

positive cells were found throughout the prostate tissue and within gland lumina. Increased CD68 positive cells were found in the prostate cancer tissue compared with benign prostate tissue (Fig. 5, compare panels A with B–D). The tissue CD68 count was increased in the prostate cancer stromal and epithelial tissue areas (average $145 \pm 53/mm^2$) compared with benign prostate ($108 \pm 31/mm^2$; Fig. 5E and Table I). The differences between these groups did not reach statistical significance using the Mann–Whitney test ($P = 0.063$). The CD68 positive cell counts were also greater in higher grade and stage prostate cancer tissue specimens, but the differences were not statistically significant (Table I).

Tissue Factor Expression in Benign and Malignant Prostate Epithelial Tissues

Prostate TF expression was determined by TF immunohistochemistry. Prostate epithelial TF expression was localized to the epithelial cell membrane and

TABLE I. Number of CD68 Positive Cells/mm^2 in Prostate Tissue

Specimen	N	Mean cell counts/mm^2 (CI)
Benign	24	108 (77–139)
Cancer	23	145 (92–278)
Gleason score		
≤6	10	140 (92–278)
7	8	143 (99–222)
≥8	5	162 (102–260)
Stage		
≤T2	16	131 (92–278)
≥T3	7	179 (111–260)

malignant gland luminal borders (Fig. 6B,C). The TF immunostaining was variable in the prostate cancer containing strongly positive and negative cells. In contrast, benign prostate epithelium showed negative or very low-TF immunostaining in almost all cases (Fig. 6A). The prostate cancer tissues showed significantly increased TF immunostaining with a digital score average of 3.4 ± 0.6 compared with benign prostate 2.8 ± 0.2 (Fig. 7A).

The pathologist manual TF score showed a similar pattern and results as the digital imaging score; however, the average pathologist determined scores for cancer of 1.9 ± 1.0 compared with 0.74 ± 0.9 for the benign prostate tissues ($P < 0.0003$). A curvilinear relationship was observed between the pathologist manual and digital image scores (Fig. 7B). A comparison of pathologist manual versus digital image scoring methods showed excellent correlation with a Spearman's correlation coefficient of 0.783 ($n = 37$, $P < 0.0001$). The TF immunostaining intensity showed no significant differences between prostate cancer grade and stage. Some prostate cancer epithelium exhibited negative TF expression, suggesting that other factors may also characterize clinically aggressive prostate tumors. Interestingly, the prostate tissue CD68 positive cell density showed a positive correlation with epithelial TF expression with a Spearman's coefficient of 0.548 ($n = 35$, $P < 0.001$).

DISCUSSION

Freshly isolated human peripheral blood monocytes as well as U-937 and THP-1 monocyte-lineage cells stimulated PC-3 and DU145 prostate cancer cell invasion activity. Several prostate cancer cell lines examined showed a positive relationship between invasion activity and TF expression. Co-culture of the prostate cancer cell lines with U-937 cells stimulated increased prostate cancer cell TF expression; however, co-culture with THP-1 cells did not produce this effect. TF neutralizing antibodies inhibited PC-3 prostate cancer cell invasion when cultured alone or in the presence of U-937 or THP-1 monocyte-lineage cells. Additional factors including cytokines, growth factors, and extracellular proteases likely contribute to U-937 and THP-1-induced PC-3 invasion. However, cancer cell TF has also been shown to play an important role in the growth, motility, and invasiveness of melanoma, hepatic, colorectal, and pancreatic tumors [27,51,52].

Clinical prostate specimens showed increased tissue CD68 positive cells in prostate cancer compared with benign prostate. Interestingly, prostate cancer epithelial TF expression was also increased and showed a very good correlation with the tissue CD68 positive cell density, which has not been previously reported. CD68 is a biomarker also known as macro-

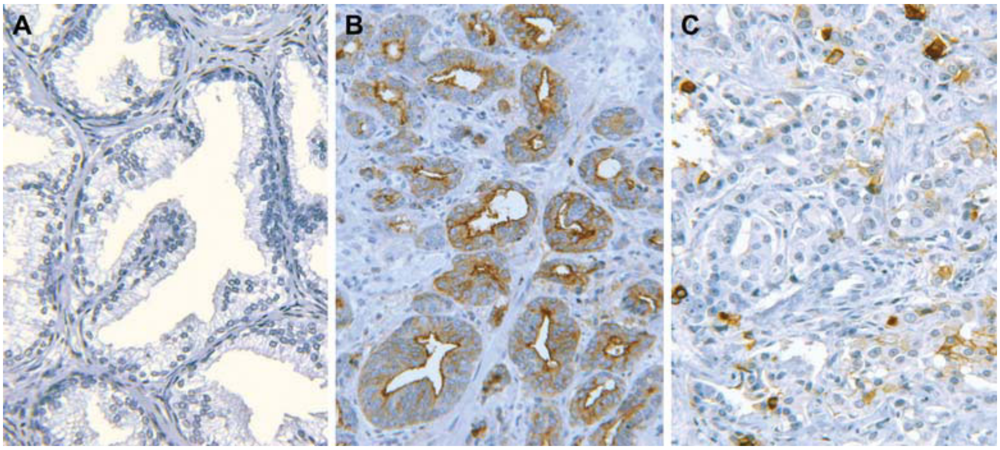

Fig. 6. Prostate tissue factor (TF) expression by immunohistochemistry. **A**: Photomicrograph showing representative TF immunostaining of benign prostate. **B**: TF immunostaining of malignant epithelial cell membrane and glandular lumina. **C**: Cancer cell TF cell membrane immunostaining.

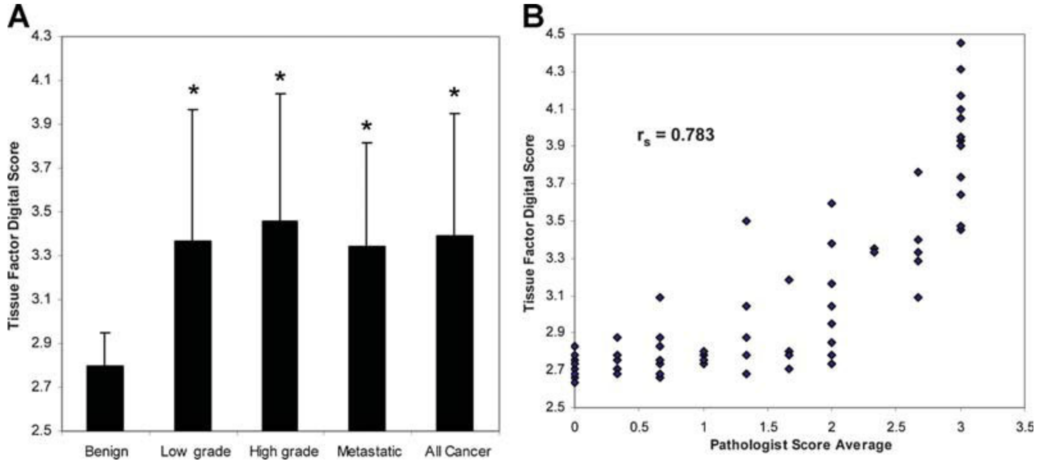

Fig. 7. Tissue factor (TF) expression scores of prostate epithelium by digital and pathologist manual methods. **A**: TF digital image quantification between prostate tissue specimen groups using ChromaVision Automatic Cellular Imaging System II software. Data expressed as mean ± SD; $*P < 0.016$. **B**: Comparison of TF immunostaining intensity between pathologist average and digital image scores.

sialin, expressed on cells of monocyte-lineage including tissue macrophages and dendritic cells. Increased tissue macrophages have also been reported in sarcomas, melanoma, and carcinomas of breast, colon, and thyroid [53–55]. TAMs may promote tumor cell growth, angiogenesis, and invasiveness through production and secretion of extracellular proteases and growth factors [54,56,57].

In vitro models provide evidence that monocytes, cytokines, and lipopolysaccharides can promote tumor cell invasion [58–61]. Co-culture with monocytes have lead to increased tumor cell invasion and increased expression of TF, matrix metalloproteinases (MMPs), urokinase-like plasminogen activator (u-PA), and nitric-oxide synthase (NOS); factors important for cancer cell invasion [60–62]. Monocyte-lineage cells and stromal cells may cross-communicate with cancer cells through several cytokines, which may recruit monocytes and promote tumor cell invasion [1,22,63–66]. Breast cancer cells have been shown to interact with monocytes to increase cancer cell TF, PAI-1, and MMP-1 expression [57]. Recent in vivo studies have shown that CSF-1 stimulated macrophages stimulated breast cancer cell invasion in a paracrine loop through EGF or heregulin that may be produced by monocytes and stromal cells in the tumor microenvironment [67,68]. Cytokines produced in the tumor microenvironment can promote tumor growth, motility, and angiogenesis through proinflammatory transcription factors including NF-κB, JNK, and AP-1 [69–71]. Interestingly, proinflammatory transcription activation through these factors also leads to increased TF expression [28,72,73]. Future studies may determine the conditions in which monocytes can stimulate prostate cancer cell TF expression.

Increased TF expression has been found in the plasma and prostate tissues of nearly 75% of patients with prostate cancer [31,74]. Increased TF expression is associated with advanced tumor grade and stage and with increased preoperative PSA levels, increased VEGF levels, and angiogenesis, pointing to TF expression as a potential risk factor for recurrent disease [31,74,75]. In addition, increased TF expression is associated with increased microvessel density in colorectal and NSCLC [30,31,38]. TF expression is also increased in aggressive melanoma, hepatic, colorectal, and pancreatic tumors [27,51,52].

Several hypotheses have been advanced on the mechanism by which TF may contribute to aggressive cancer behavior. It is postulated that TF may stimulate cell growth and migration through receptor ligation or generation of downstream proteases such as thrombin, which activate cells through protease-activated receptors (PARs) [76–78]. In addition, tumor cells that express TF may promote an inflammatory response and malignant progression [40,76]. TF also stimulates thrombin generation, fibrin polymerization, and formation of a provisional fibrin matrix. These processes may work together to promote tumor growth and spread [41,42].

This study demonstrates that monocyte-lineage cells stimulated increased prostate cancer cell invasion. Highly invasive prostate cancer cells exhibited increased TF expression, which was required, at least in part, for optimal invasion activity. Clinical prostate cancer tissues showed increased epithelial TF expression and increased CD68 positive cells when compared with benign prostate. Further studies are needed to determine how the tumor microenvironment and monocytes may contribute to increased prostate

cancer TF expression and aggressive prostate cancer behavior.

ACKNOWLEDGMENTS

We thank Dr. James Morrissey and Dr. Gerald Soff for the gift of Tissue Factor antibodies. We thank Dr. Chung Lee and Dr. Susan Crawford for helpful discussions and suggestions. This study was funded in part by the Department of Defense New Investigator Grant DAMD17-02-1-0162 and a Research Career Development Award from the National Cancer Institute Prostate S.P.O.R.E. P50-CA090386 at Northwestern University Robert H. Lurie Comprehensive Cancer Center.

REFERENCES

1. Karan D, Holzbeierlein J, Thrasher JB. Macrophage inhibitory cytokine-1: Possible bridge molecule of inflammation and prostate cancer. Cancer Res 2009; 69(1): 2–5.
2. Yu JL, Rak JW. Host microenvironment in breast cancer development: Inflammatory and immune cells in tumour angiogenesis and arteriogenesis. Br Cancer Res 2003; 5(2): 83–88.
3. Torisu H, Ono M, Kiryu H, Furue M, Ohmoto Y, Nakayama J, Nishioka Y, Sone S, Kuwano M. Macrophage infiltration correlates with tumor stage and angiogenesis in human malignant melanoma: Possible involvement of TNFalpha and IL-1alpha. Int J Cancer 2000; 85(2): 182–188.
4. Balkwill F, Mantovani A. Inflammation and cancer: Back to Virchow? Lancet 2001; 357(9255): 539–545.
5. Kuper H, Adami HO, Trichopoulos D. Infections as a major preventable cause of human cancer. J Intern Med 2000; 248(3): 171–183.
6. Coussens LM, Werb Z. Inflammation and cancer. Nature 2002; 420(6917): 860–867.
7. Ernst PB, Gold BD. The disease spectrum of *Helicobacter pylori*: The immunopathogenesis of gastroduodenal ulcer and gastric cancer. Ann Rev Microbiol 2000; 54: 615–640.
8. Shacter E, Weitzman SA. Chronic inflammation and cancer. Oncology 2002; 16(2): 217–226.
9. Hayes RB, Pottern LM, Strickler H, Rabkin C, Pope V, Swanson GM, Greenberg RS, Schoenberg JB, Liff J, Schwartz AG, Hoover RN, Fraumeni JF Jr. Sexual behaviour, STDs and risks for prostate cancer. Br J Cancer 2000; 82(3): 718–725.
10. De Marzo AM, Meeker AK, Zha S, Luo J, Nakayama M, Platz EA, Isaacs WB, Nelson WG. Human prostate cancer precursors and pathobiology. Urology 2003; 62(5 Supplement 1): 55–62.
11. Platz EA, De Marzo AM. Epidemiology of inflammation and prostate cancer. J Urology 2004; 171(2 Pt 2): S36–S40.
12. De Marzo AM, Nakai Y, Nelson WG, Inflammation, atrophy, and prostate carcinogenesis. Urology 2007; 25(5): 398–400.
13. Liotta LA, Kohn EC. The microenvironment of the tumour-host interface. Nature 2001; 411(6835): 375–379.
14. Zheng SL, Augustsson-Balter K, Chang B, Hedelin M, Li L, Adami HO, Bensen J, Li G, Johnasson JE, Turner AR, Adams TS, Meyers DA, Isaacs WB, Xu J, Gronberg H. Sequence variants of toll-like receptor 4 are associated with prostate cancer risk: Results from the CAncer Prostate in Sweden Study. Cancer Res 2004; 64(8): 2918–2922.
15. Blum DL, Koyama T, M'Koma AE, Iturregui JM, Martinez-Ferrer M, Uwamariya C, Smith JA Jr, Clark PE, Bhowmick NA. Chemokine markers predict biochemical recurrence of prostate cancer following prostatectomy. Clin Cancer Res 2008; 14(23): 7790–7797.
16. Allavena P, Sica A, Solinas G, Porta C, Mantovani A. The inflammatory micro-environment in tumor progression: The role of tumor-associated macrophages. Crit Rev Oncol Hematol 2008; 66(1): 1–9.
17. Marcus B, Arenberg D, Lee J, Kleer C, Chepeha DB, Schmalbach CE, Islam M, Paul S, Pan Q, Hanash S, Kuick R, Merajver SD, Teknos TN. Prognostic factors in oral cavity and oropharyngeal squamous cell carcinoma. Cancer 2004; 101(12): 2779–2787.
18. Liu S-Y, Chang L-C, Pan L-F, Hung Y-J, Lee C-H, Shieh Y-S. Clinicopathologic significance of tumor cell-lined vessel and microenvironment in oral squamous cell carcinoma. Oral Oncol 2008; 44(3): 277–285.
19. Moser PL, Brunner A, Horninger W, Bartsch G, Mikuz G. Correlation between inflammatory cells (T and B lymphocytes, macrophages) in prostate biopsies and elevated PSA levels in a PSA screening population. Urology 2002; 59(1): 68–72.
20. Yang G, Addai J, Tian W-H, Frolov A, Wheeler TM, Thompson TC. Reduced infiltration of class A scavenger receptor positive antigen-presenting cells is associated with prostate cancer progression. Cancer Res 2004; 64(6): 2076–2082.
21. Mantovani A. Tumor-associated macrophages in neoplastic progression: A paradigm for the in vivo function of chemokines. Lab Invest 1994; 71(1): 5–16.
22. Reiland J, Furcht LT, McCarthy JB. CXC-chemokines stimulate invasion and chemotaxis in prostate carcinoma cells through the CXCR2 receptor. Prostate 1999; 41(2): 78–88.
23. Wang W, Bergh A, Damber JE. Cyclooxygenase-2 expression correlates with local chronic inflammation and tumor neovascularization in human prostate cancer. Clin Cancer Res 2005; 11(9): 3250–3256.
24. Chen JJ, Lin YC, Yao PL, Yuan A, Chen HY, Shun CT, Tsai MF, Chen CH, Yang PC. Tumor-associated macrophages: The double-edged sword in cancer progression. J Clin Oncol 2005; 23(5): 953–964.
25. Greten FR, Eckmann L, Greten TF, Park JM, Li ZW, Egan LJ, Kagnoff MF, Karin M. IKKbeta links inflammation and tumorigenesis in a mouse model of colitis-associated cancer. Cell 2004; 118(3): 285–296.
26. Luo JL, Maeda S, Hsu LC, Yagita H, Karin M. Inhibition of NF-kappaB in cancer cells converts inflammation-induced tumor growth mediated by TNFalpha to TRAIL-mediated tumor regression. Cancer Cell 2004; 6(3): 297–305.
27. Bromberg ME, Sundaram R, Homer RJ, Garen A, Konigsberg WH. Role of tissue factor in metastasis: Functions of the cytoplasmic and extracellular domains of the molecule. Thromb Haemost 1999; 82(1): 88–92.
28. Oeth P, Parry GC, Mackman N. Regulation of the tissue factor gene in human monocytic cells. Role of AP-1, NF-kappa B/Rel, and Sp1 proteins in uninduced and lipopolysaccharide-induced expression. Arterioscler ThrombVasc Biol 1997; 17(2): 365–374.
29. Nakasaki T, Wada H, Shigemori C, Miki C, Gabazza EC, Nobori T, Nakamura S, Shiku H. Expression of tissue factor and vascular endothelial growth factor is associated with angiogenesis in colorectal cancer. Am J Hematol 2002; 69(4): 247–254.
30. Koomagi R, Volm M. Tissue-factor expression in human non-small cell lung carcinoma measured by immunohistochemistry: Correlation between tissue factor and angiogenesis. Int J Cancer 1998; 79(1): 19–22.

31. Abdulkadir SA, Carvalhal GF, Kaleem Z, Kisiel W, Humphrey PA, Catalona WJ, Milbrandt J. Tissue factor expression and angiogenesis in human prostate carcinoma. Hum Pathol 2000; 31(4): 443–447.
32. Akashi T, Furuya Y, Ohta S, Fuse H. Tissue factor expression and prognosis in patients with metastatic prostate cancer. Urology 2003; 62(6): 1078–1082.
33. Martin DM, Boys CW, Ruf W. Tissue factor: Molecular recognition and cofactor function. FASEB J 1995; 9(10): 852–859.
34. Hillen HF. Thrombosis in cancer patients. Ann Oncol 2000; 11(Suppl 3): 273–276.
35. Lykke J, Nielsen HJ. The role of tissue factor in colorectal cancer. Eur J Surg Oncol 2003; 29(5): 417–422.
36. Shigemori C, Wada H, Matsumoto K, Shiku H, Nakamura S, Suzuki H. Tissue factor expression and metastatic potential of colorectal cancer. Thromb Haemost 1998; 80(6): 894–898.
37. Sawada M, Miyake S, Ohdama S, Matsubara O, Masuda S, Yakumaru K, Yoshizawa Y. Expression of tissue factor in non-small cell lung cancers and its relationship to metastasis. Br J Cancer 1999; 79(3-4): 472–477.
38. Nakasaki T, Wada H, Shigemori C, Miki C, Gabazza EC, Nobori T, Nakamura S, Shiku H. Expression of tissue factor and vascular endothelial growth factor is associated with angiogenesis in colorectal cancer. Am J Hematol 2002; 69(4): 247–254.
39. Bromberg ME, Konigsberg WH, Madison JF, Pawashe A, Garen A. Tissue factor promotes melanoma metastasis by a pathway independent of blood coagulation. Proc Natl Acad Sci USA 1995; 92(18): 8205–8209.
40. Yu JL, May L, Klement P, Weitz JI, Rak J. Oncogenes as regulators of tissue factor expression in cancer: Implications for tumor angiogenesis and anti-cancer therapy. Semin Thromb Hemost 2004; 30(1): 21–30.
41. Fernandez PM, Patierno SR, Rickles FR. Tissue factor and fibrin in tumor angiogenesis. Semin Thromb Hemost 2004; 30(1): 31–44.
42. Rickles FR, Patierno S, Fernandez PM. Tissue factor, thrombin, and cancer. Chest 2003; 124(3 suppl.): 58S–68S.
43. Lindholm PF, Bub J, Kaul S, Shidham VB, Kajdacsy-Balla A. The role of constitutive NF-κB activity in PC-3 human prostate cancer cell invasive behavior. Clin Exp Metastasis 2001; 18(6): 471–479.
44. Hodge JC, Bub J, Kaul S, Kajdacsy-Balla A, Lindholm PF. Requirement of RhoA activity for increased nuclear factor kappaB activity and PC-3 human prostate cancer cell invasion. Cancer Res 2003; 63(6): 1359–1364.
45. Repesh LA. A new in vitro assay for quantitating tumor cell invasion. Invasion Metastasis 1989; 9(3): 192–208.
46. Hwang YS, Hodge JC, Sivapurapu N, Lindholm PF. Lysophosphatidic acid stimulates PC-3 prostate cancer cell Matrigel invasion through activation of RhoA and NF-kB activity. Mol Carcinog 2006; 45(7): 518–529.
47. Wahl LM, Wahl SM, Smythies LE, Smith PD. Immunologic studies in humans. In: Coligan JE, Kruisbeek AM, Margulies DH, Shevach EM, editors. Unit 7.6A isolation of monocyte/macrophage populations. John Wiley; 2005. 7.6.1–7.6.3.
48. Bloom K, Harrington D. Enhanced accuracy and reliability of HER-2/neu immunohistochemical scoring using digital microscopy. Am J Clin Pathol 2004; 121(5): 620–630.
49. Ellis CM, Dyson MJ, Stephenson TJ, Maltby EL. HER2 amplification status in breast cancer: A comparison between immunohistochemical staining and fluorescence in situ hybridisation using manual and automated quantitative image analysis scoring techniques. J Clin Pathol 2005; 58(7): 710–714.
50. Lee CM, Lee RJ, Hammond E, Tsodikov A, Dodson M, Zempolich K, Gaffney DK. Expression of HER2neu (c-erbB-2) and epidermal growth factor receptor in cervical cancer: Prognostic correlation with clinical characteristics, and comparison of manual and automated imaging analysis. Gynecol Oncol 2004; 93(1): 209–214.
51. Seto S, Onodera H, Kaido T, Yoshikawa A, Ishigami S, Arii S, Imamura M. Tissue factor expression in human colorectal carcinoma: Correlation with hepatic metastasis and impact on prognosis. Cancer 2000; 88(2): 295–301.
52. Kakkar AK, Chinswangwatanakul V, Lemoine NR, Tebbutt S, Williamson RC. Role of tissue factor expression on tumour cell invasion and growth of experimental pancreatic adenocarcinoma. Br J Surg 1999; 86(7): 890–894.
53. Ryder M, Ghossein RA, Ricarte-Filho JCM, Knauf JA, Fagin JA. Increased density of tumor-associated macrophages is associated with decreased survival in advanced thyroid cancer. Endocr Relat Cancer 2008; 15(4): 1069–1074.
54. Pollard JW. Macrophages define the invasive microenvironment in breast cancer. J Leukocyte Biol 2008; 84(3): 623–630.
55. Allavena P, Garlanda C, Borrello MG, Sica A, Mantovani A. Pathways connecting inflammation and cancer. Curr Opin Genet Dev 2008; 18(1): 3–10.
56. Lissbrant IF, Stattin P, Wikstrom P, Damber JE, Egevad L, Bergh A. Tumor associated macrophages in human prostate cancer: Relation to clinicopathological variables and survival. Int J Oncol 2000; 17(3): 445–451.
57. Blot E, Chen W, Vasse M, Paysant J, Denoyelle C, Pille JY, Vincent L, Vannier JP, Soria J, Soria C. Cooperation between monocytes and breast cancer cells promotes factors involved in cancer aggressiveness. Br J Cancer 2003; 88(8): 1207–1212.
58. Mukai M, Shinkai K, Tateishi R, Mori Y, Akedo H. Macrophage potentiation of invasive capacity of rat ascites hepatoma cells. Cancer Res 1987; 47(8): 2167–2171.
59. Jiang WG, Hiscox S, Hallett MB, Mansel RE, Puntis MC. Regulation of motility and invasion of cancer cells by human monocytic cells. Anticancer Res 1995; 15(4): 1303–1310.
60. Hagemann T, Robinson SC, Schulz M, Trumper L, Balkwill FR, Binder C. Enhanced invasiveness of breast cancer cell lines upon co-cultivation with macrophages is due to TNF-alpha dependent up-regulation of matrix metalloproteases. Carcinogenesis 2004; 25(8): 1543–1549.
61. Siegert A, Rosenberg C, Schmitt WD, Denkert C, Hauptmann S. Nitric oxide of human colorectal adenocarcinoma cell lines promotes tumour cell invasion. Br J Cancer 2002; 86(8): 1310–1315.
62. Blot E, Chen W, Vasse M, Paysant J, Denoyelle C, Pille JY, Vincent L, Vannier JP, Soria J, Soria C. Cooperation between monocytes and breast cancer cells promotes factors involved in cancer aggressiveness. Br J Cancer 2003; 88(8): 1207–1212.
63. Karan D, Thrasher JB, Lubaroff D. Prostate cancer: Genes, environment, immunity and the use of immunotherapy. Prostate Cancer Prostatic Dis 2008; 11(3): 230–236.
64. Richardsen E, Uglehus RD, Due J, Busch C, Busund LTR. The prognostic impact of M-CSF, CSF-1 receptor, CD68 and CD3 in prostatic carcinoma. Histopathology 2008; 53(1): 30–38.
65. Mazzucchelli L, Loetscher P, Kappeler A, Uguccioni M, Baggiolini M, Laissue JA, Mueller C. Monocyte chemoattractant protein-1 gene expression in prostatic hyperplasia and prostate adenocarcinoma. Am J Pathol 1996; 149(2): 501–509.
66. Waugh DJJ, Wilson C. The interleukin-8 pathway in cancer. Clin Cancer Res 2008; 14(21): 6735–6741.

67. Hernandez L, Smirnova T, Kedrin D, Wyckoff J, Zhu L, Stanley ER, Cox D, Muller WJ, Pollard JW, Van Rooijen N, Segall JE. The EGF/CSF-1 paracrine invasion loop can be triggered by heregulin beta1 and CXCL12. Cancer Res 2009; 69(7): 3221–3227.
68. Wyckoff J, Wang W, Lin EY, Wang Y, Pixley F, Stanley ER, Graf T, Pollard JW, Segall J, Condeelis J. A paracrine loop between tumor cells and macrophages is required for tumor cell migration in mammary tumors. Cancer Res 2004; 64(19): 7022–7029.
69. Ono M. Molecular links between tumor angiogenesis and inflammation: Inflammatory stimuli of macrophages and cancer cells as targets for therapeutic strategy. Cancer Sci 2008; 99(8): 1501–1506.
70. Hagemann T, Wilson J, Kulbe H, Li NF, Leinster DA, Charles K, Klemm F, Pukrop T, Binder C, Balkwill FR. Macrophages induce invasiveness of epithelial cancer cells via NF-kappa B and JNK. J Immunol 2005; 175(2): 1197–1205.
71. Harkonen PL, Vaananen HK. Monocyte-macrophage system as a target for estrogen and selective estrogen receptor modulators. Ann NY Acad Sci 2006; 1089: 218–227.
72. Napoleone E, di Santo A, Peri G, Mantovani A, de Gaetano G, Donati MB, Lorenzet R. The long pentraxin PTX3 up-regulates tissue factor in activated monocytes: Another link between inflammation and clotting activation. J Leukocyte Biol 2004; 76(1): 203–209.
73. Hall AJ, Vos HL, Bertina RM. Lipopolysaccharide induction of tissue factor in THP-1 cells involves Jun protein phosphorylation and nuclear factor kappaB nuclear translocation. J Biol Chem 1999; 274(1): 376–383.
74. Langer F, Chun FK-H, Amirkhosravi A, Friedrich M, Leuenroth S, Eifrig B, Bokemeyer C, Francis JL. Plasma tissue factor antigen in localized prostate cancer: Distribution, clinical significance and correlation with haemostatic activation markers[see comment]. Thromb Haemost 2007; 97(3): 464–470.
75. Kaushal V, Mukunyadzi P, Siegel ER, Dennis RA, Johnson DE, Kohli M. Expression of tissue factor in prostate cancer correlates with malignant phenotype. Appl Immunohistochem Mol Morphol 2008; 16(1): 1–6.
76. Bromberg ME, Bailly MA, Konigsberg WH. Role of protease-activated receptor 1 in tumor metastasis promoted by tissue factor. Thromb Haemost 2001; 86(5): 1210–1214.
77. Jiang X, Bailly MA, Panetti TS, Cappello M, Konigsberg WH, Bromberg ME. Formation of tissue factor-factor VIIa-factor Xa complex promotes cellular signaling and migration of human breast cancer cells. Thromb Haemost 2004; 2(1): 93–101.
78. Hjortoe GM, Petersen LC, Albrektsen T, Sorensen BB, Norby PL, Mandal SK, Pendurthi UR, Rao LV. Tissue factor-factor VIIa-specific up-regulation of IL-8 expression in MDA-MB-231 cells is mediated by PAR-2 and results in increased cell migration. Blood 2004; 103(8): 3029–3037.
