# Supplementary material for: Monocyte-Induced Prostate Cancer Cell Invasion is Mediated by Chemokine ligand 2 and Nuclear Factor-κB Activity
Source: J Clin Cell Immunol. Author manuscript; Available in PMC 2015 Aug 25. (PMC4548876; doi:10.4172/2155-9899.1000308)
Supplement: Supplementary file [file NIHMS699109-supplement-Supplementary_file.zip › NF-kB_mediated_TGF-b_Induced Vimentin_Expression_Bichemical_Recurrence_ClinCR_2009.pdf]

DOI:10.1158/1078-0432.CCR-08-1656

Clinical Cancer Research

Nuclear Factor-κB-Mediated Transforming Growth Factor-β-Induced Expression of Vimentin Is an Independent Predictor of Biochemical Recurrence after Radical Prostatectomy

Qiang Zhang, Brian T. Helfand, Thomas L. Jang, et al.

Clin Cancer Res 2009;15:3557-3567. Published online May 15, 2009.

**Updated Version**	Access the most recent version of this article at: doi:10.1158/1078-0432.CCR-08-1656
**Supplementary Material**	Access the most recent supplemental material at: http://clincancerres.aacrjournals.org/content/suppl/2009/05/20/1078-0432.CCR-08-1656.DC1.html

**Cited Articles**	This article cites 34 articles, 12 of which you can access for free at: http://clincancerres.aacrjournals.org/content/15/10/3557.full.html#ref-list-1
**Citing Articles**	This article has been cited by 4 HighWire-hosted articles. Access the articles at: http://clincancerres.aacrjournals.org/content/15/10/3557.full.html#related-urls

**E-mail alerts**	Sign up to receive free email-alerts related to this article or journal.
**Reprints and Subscriptions**	To order reprints of this article or to subscribe to the journal, contact the AACR Publications Department at pubs@aacr.org.
**Permissions**	To request permission to re-use all or part of this article, contact the AACR Publications Department at permissions@aacr.org.

Downloaded from clincancerres.aacrjournals.org on May 21, 2012
Copyright © 2009 American Association for Cancer Research

DOI:10.1158/1078-0432.CCR-08-1656

Nuclear Factor-κB-Mediated Transforming Growth Factor-β-Induced Expression of Vimentin Is an Independent Predictor of Biochemical Recurrence after Radical Prostatectomy

Qiang Zhang,[1,5] Brian T. Helfand,[1] Thomas L. Jang,[6] Lihua J. Zhu,[7] Lin Chen,[2] Ximing J. Yang,[3,5] James Kozlowski,[1,5] Norm Smith,[1,5] Shilajit D. Kundu,[6] Guangyu Yang,[3,5] Adekunle A. Raji,[3] Borko Javonovic,[4,5] Michael Pins,[3,5] Paul Lindholm,[3,5] Yinglu Guo,[8] William J. Catalona,[1,5] and Chung Lee[1,5]

Abstract

Purpose: Transforming growth factor-β (TGF-β)-mediated epithelial-to-mesenchymal transition (EMT) has been shown to occur in some cancers; however, the pathway remains controversial and varies with different cancers. In addition, the mechanisms by which TGF-β and the EMT contribute to prostate cancer recurrence are largely unknown. In this study, we elucidated TGF-β-mediated EMT as a predictor of disease recurrence after therapy for prostate cancer, which has not been reported before.

Experimental Design: We analyzed TGF-β-induced EMT using nuclear factor-κB (NF-κB) as an intermediate mediator in prostate cancer cell lines. A total of 287 radical prostatectomy specimens were evaluated using immunohistochemistry in a high-throughput tissue microarray analysis. Levels of TGF-β signaling components and EMT-related factors were analyzed using specific antibodies. Results were expressed as the percentage of cancer cells that stained positive for a given antibody and were correlated with disease recurrence rates at a mean of 7 years following radical prostatectomy.

Results: In prostate cancer cell lines, TGF-β-induced EMT was mediated by NF-κB signaling. Blockade of NF-κB or TGF-β signaling resulted in abrogation of vimentin expression and inhibition of the invasive capability of these cells. There was high risk of biochemical recurrence associated with tumors that displayed high levels of expression of TGF-β1, vimentin, and NF-κB and low level of cytokeratin 18. This was particularly true for vimentin, which is independent of patients' Gleason score.

Conclusions: The detection of NF-κB-mediated TGF-β-induced EMT in primary tumors predicts disease recurrence in prostate cancer patients following radical prostatectomy. The changes in TGF-β signaling and EMT-related factors provide novel molecular markers that may predict prostate cancer outcomes following treatment.

Prostate cancer is the most common malignancy in American males and is second only to lung cancer as the leading cause of cancer-specific mortality (1). Metastases are responsible for mortality in prostate cancer patients. Post-treatment serum prostate-specific antigen (PSA) values have been used to identify patients at risk for metastases. In fact, depending on the definition used, the 5-year PSA recurrence rates following either radical prostatectomy or radiation therapy were reported to be up to 31%, and the 10-year clinical recurrence rates in these patients were reported to be ~75% (2–4). A short time interval to biochemical recurrence, rapid PSA doubling times, and high Gleason scores are all considered high-risk factors for prostate cancer-specific mortality (5, 6). However, these clinical characteristics have not been proven to be useful predictors of clinical outcome in patients with low-grade disease (Gleason score ≤6; refs. 7–9). Therefore,

Authors' Affiliations: Departments of [1]Urology, [2]Ophthalmology, [3]Pathology, and [4]Preventive Medicine, Northwestern University Feinberg School of Medicine; [5]Robert H. Lurie Comprehensive Cancer Center, Northwestern University, Chicago, Illinois; [6]Memorial Sloan-Kettering Cancer Center, New York, New York; [7]Program in Gene Function and Expression, University of Massachusetts Medical School, Worcester, Massachusetts; and [8]Institute of Urology, The First Hospital, Peking University, Beijing, China

Received 6/27/08; revised 1/30/09; accepted 2/3/09.

Grant support: American Cancer Society (Illinois) grant 08-22; American Cancer Society institutional research grant ACS-IRG 93-037-12; American Urological Association Foundation; Department of Defense grants PC970410, PC001491, and PC030038; National Cancer Institute grants CA90386, CA107186, CA114810; Illinois Department of Public Health contract 83284034; and a gift from Mr. Fred L. Turner.

The costs of publication of this article were defrayed in part by the payment of page charges. This article must therefore be hereby marked *advertisement* in accordance with 18 U.S.C. Section 1734 solely to indicate this fact.

Note: Supplementary data for this article are available at Clinical Cancer Research Online (http://clincancerres.aacrjournals.org/).

Requests for reprints: Chung Lee or Qiang Zhang, Department of Urology, Northwestern University Feinberg School of Medicine, 303 East Chicago Avenue, Tarry 16-733, Chicago, IL 60611. Phone: 312-908-2004; Fax: 312-908-7275; E-mail: c-lee7@northwestern.edu or q-zhang2@northwestern.edu.

© 2009 American Association for Cancer Research.

doi:10.1158/1078-0432.CCR-08-1656

Downloaded from clincancerres.aacrjournals.org on May 21, 2012
Copyright © 2009 American Association for Cancer Research

DOI:10.1158/1078-0432.CCR-08-1656

Translational Relevance

Cancer tissues obtained at the time of radical prostatectomy contain a wealth of information, which may predict the future fate of the disease. Yet, a suitable marker to predict disease recurrence has not been available in prostate cancer. Transforming growth factor-β (TGF-β) is an important regulator in cancer progression. In this exploratory study, we describe a novel method based on tumor-derived, TGF-β-induced expression of vimentin to evaluate the recurrence of prostate cancer after surgical treatment. Our findings indicate that high levels of vimentin expression in prostatectomy specimens may independently predict disease recurrence in prostate cancer patients. The markers involved in TGF-β-induced epithelial-to-mesenchymal transition, such as TGF-β, nuclear factor-κB, vimentin, and cytokeratin 18, might be applied to the evaluation or prediction of the outcome following the treatment of radical prostatectomy in clinic.

additional biomarkers are needed to predict recurrence after radical prostatectomy.

Biomarkers expressed in each tumor reflect the clinical signature of the disease and may determine the clinical course. Although other methods of metastases have recently been described, the epithelial-to-mesenchymal transition (EMT) has been considered one of the major mechanisms mediating invasion and metastasis of cancer cells (10). The role of transforming growth factor-β (TGF-β) signaling in EMT has been established for many normal and transformed cell lines (11, 12). Recently, the expression of vimentin has been linked to the EMT in prostate cancer and metastasis *in vitro* (13). Similarly, increased vimentin expression levels have been associated with poorly differentiated prostate cancer (14). Although TGF-β is implicated in invasion in prostate cancer cell invasion by up-regulating vimentin expression (15), the precise mechanism and downstream mediators remain incompletely defined (16). From an intracellular signaling perspective, TGF-β may induce the EMT and invasion via a Smad-dependent pathway by activating Jag1 and Hey1 (17). There is evidence to indicate that other intracellular signaling pathways also play a role in EMT (16). For example, nuclear factor-κB (NF-κB) may be activated by TGF-β signaling through activation of TAK1 (11). Also, Ras may act with TGF-β to activate NF-κB (18). However, although many of these TGF-β-interacting proteins have been associated with the EMT and invasion, they have not specifically been implicated in prostate cancer metastasis. Therefore, the purpose of the present study is 2-fold: (*a*) to elucidate the mechanisms underlying TGF-β-induced EMT and (*b*) to explore factors involved in this pathway as potential markers for evaluating biochemical recurrence in prostate cancer patients following radical prostatectomy.

Materials and Methods

Prostate cancer cell lines. Four variants of human prostate cancer PC-3 cell lines were kindly provided by Drs. Fidler and Pettaway of the M. D. Anderson Cancer Center (19). These variants included PC-3 (wild type), PC-3M-Pr04 (selected for cancer cells confined to the prostate), PC-3M (selected for metastasis), and PC-3M-LN4 (selected for metastasis to lymph nodes). These variants are ideal for studying the role of TGF-β and NF-κB in tumor migration and invasion. PC-3 and PC-3M-Pr04 are less aggressive than PC-3M and PC-3M-LN4. As a negative control, cells were rendered insensitive to TGF-β by introducing a dominant-negative TGF-β type II receptor vector (TβRIIDN; ref. 20; Fig. 1A, *top right*). The infection efficiency was >95.9% for the TβRIIDN vector. Cells were cultured in RPMI 1640 (Life Technologies) supplemented with 10% heat-inactivated fetal bovine serum (Life Technologies). Cells were treated with or without TGF-β (10 ng/mL, 24 h). A NF-κB inhibitor, BAY11-7085 (*E*)-3-[(4-*t*-butylphenylsulfony1)]-2-propenenitrile (BIOMOL Research Laboratories), was used at 20 μmol/L, 24 h.

TGF-β1 ELISA. All variants (PC-3, PC-3M-Pr04, PC-3M-LN4, and PC-3M) and the corresponding TβRIIDN-transfected cell lines were cultured in fresh serum-free medium for 24 h (1.0×10^7 per T75 flask). The pooled conditioned medium was collected and concentrated by using YM-3 Centriprep Centrifugal Filter Devices (Millipore). TGF-β1 ELISA was carried out using the Quantikine Human TGF-β1 Immunoassay Kit from R&D Systems. The total number of cells in each flask was counted using a Coulter counter and the levels of TGF-β1 were reported as pg/10^5 cells/48 h.

[^{3}H]thymidine incorporation assay. All cells were grown in culture for 48 h. Cells were then exposed to a medium containing [^{3}H]thymidine (0.5 μCi/mL; Amersham Biosciences) for an additional 5 h. The experiment was terminated by washing with warm serum-free medium. NaOH (0.1 mol/L) was added to all cell culture wells (1 mL). An aliquot of 100 μL was removed for measurement of the protein content and the remainder was used for the determination of the radioactivity as in counts/min. Thymidine incorporation was expressed as the fraction of counts found in cells of untreated controls.

Western blot analysis. Cell lysates were prepared by adding lysis buffer (50 mmol/L Tris-HCl, 1% NP-40, 0.25% sodium deoxycholate, 150 mmol/L NaCl, 1 mmol/L EDTA, 1 mmol/L Na_3VO_4, and 1 mmol/L NaF) to cell pellets. An aliquot of ~30 μg total protein was loaded onto 10% acrylamide gel in Tris-HCl (Bio-Rad). Electrophoresis was carried out in Tris-glycine-SDS running buffer and transferred to a polyvinylidene difluoride membrane. Blots were probed for the total and phosphorylated Smad2 (Santa Cruz Biotechnology) at 1:500, vimentin at 1:500 (DAKO), and cytokeratin 18 (CK18; DAKO) at 1:500 and then stripped and reprobed for glyceraldehyde-3-phosphate dehydrogenase (Advanced) with a monoclonal antibody at 1:300. Proteins of interest were detected with the enhanced chemiluminescence detection kit (Amersham Biosciences) followed by exposure to Kodak X-OMAT AR film.

Immunofluorescence and microscopy. Immunofluorescent studies were done on all PC-3 variants as described previously (21). For colocalization of vimentin and NF-κB, cells were analyzed by using nucleus-vimentin-NF-κB triple staining. For colocalization of TβRIIDN (with GFP) and vimentin or NF-κB, cells were analyzed by nucleus-vimentin or nucleus-NF-κB double staining. All slides were deparaffinized and blocked by normal serum. Stained slides were viewed with Nikon TE2000-U fluorescent microscopy (Nikon). Images were digitized by Photoshop 7.0 with a PC computer.

Cell invasion assay. Cell invasion assay (Matrigel invasion assay) was done in a 24-well Transwell chamber (8 μm pore size; CytoSelect; Cell Biolabs). Cells were plated at a density of 0.5×10^6 to 1.0×10^6/mL in serum-free medium. TGF-β1 and/or BAY11-7085 were added directly to the cell suspension, and 24 h later, the suspension was aspirated and the invaded cells were counted with a light microscope under high magnification objective (×100; Olympus) and measured at $A_{560\ nm}$ in a plate reader after treatment with the extraction solution.

Construction of tissue microarrays and clinical outcome assessment. A series of prostate tissue microarray was constructed with formalin-fixed, paraffin-embedded prostatectomy specimens as described previously (22). At least three cores were taken from each donor tissue block.

Downloaded from clincancerres.aacrjournals.org on May 21, 2012
Copyright © 2009 American Association for Cancer Research

DOI:10.1158/1078-0432.CCR-08-1656

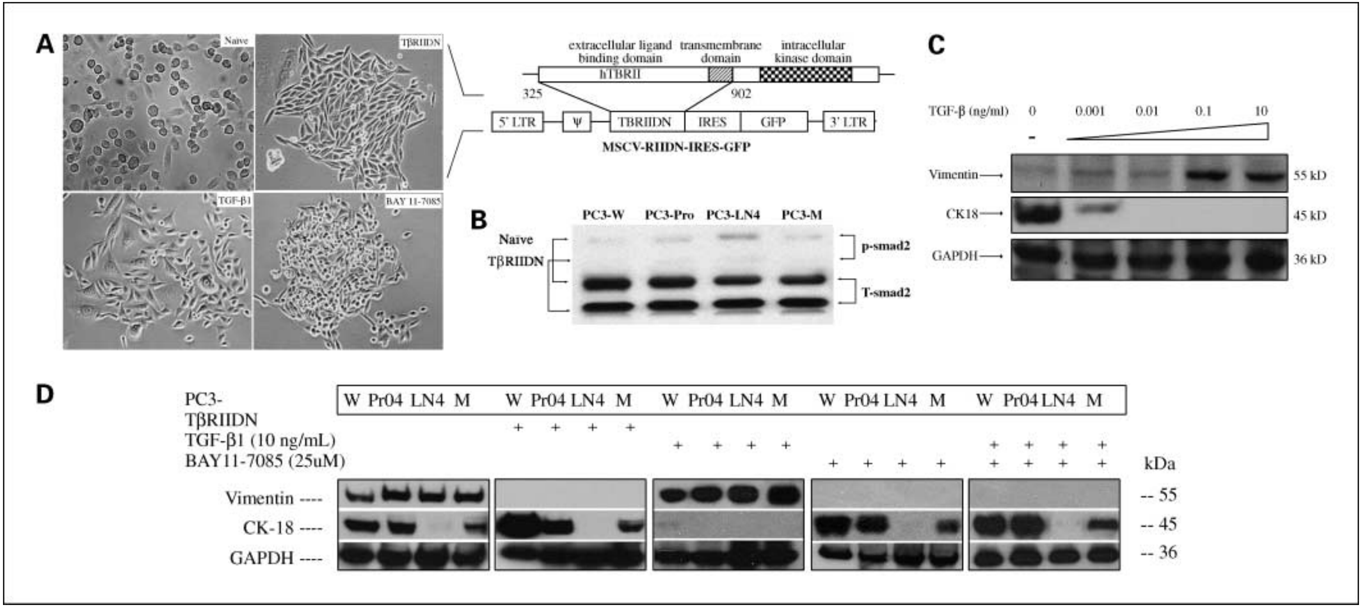

Fig. 1. NF-κB-mediated TGF-β-induced up-regulation of vimentin and down-regulation of CK18 in prostate cancer. *A, left,* overall morphology of prostate cancer cells (with PC-3 as sample, the cells were plated for 72 h; the confluent percentage is 20% at the beginning) shape changed and showed an extended and elongated shape, and cell-cell contact decreased after treatment with TGF-β. Meanwhile, after down-regulating TGF-β signal by infection with a TβRIIDN or treatment by BAY11-7085, all cells, irrespective of their aggressive potential, recovered cell-cell contact (magnification, 10 × 10). *Right,* construct of TβRIIDN retrovirus vector. *B,* Western blot showed that, after infection of TβRIIDN, the activity of p-Smad2 of all PC-3, PC-3M-Pr04, PC-3M-LN4, and PC-3M was down-regulated significantly. *C,* there is a dose-dependent relationship between treatment of TGF-β and expression of vimentin (positive) and CK18 (reverse) in all PC-3 cell lines (with PC-3 as sample, the amount of protein loaded is 8.0 μg). *D,* high level of vimentin expression and much lower CK18 expression levels are found in PC-3M and PC-3M-LN4, respectively, which have more aggressive potential compared with PC-3. After blocking TGF-β by infection with a TβRIIDN, vimentin expression was down-regulated in each group and CK18 was up-regulated correspondingly, except for PC-3M-LN4. Interestingly, on treatment with NF-κB inhibitor, the effect of TGF-β on EMT was reversed. The detailed graph could be found in Supplementary Fig. S2. This result showed that NF-κB is the major mediator downstream for TGF-β-induced EMT in prostate cancer cell lines (to distinguish the expression between each PC-3 variants, we loaded 20 μg protein for each sample).

A total of 287 radical prostatectomy specimens were obtained from the Prostate SPORE Tissue Bank at Northwestern University. All patients (n = 287) for whom there were tissue microarray samples were included in the study. Power calculation using the pwr.r.test function of PWR package in R showed that a sample size of 287 patients provides 99% power to detect a correlation with correlation coefficient of 0.25 (or -0.25) at the significance level of 0.05.

All enrolled subjects provided written informed consent and the study was approved by the institutional review board of Northwestern University. These specimens included 165 cases with low Gleason score (≤6), 55 cases with intermediate Gleason score (7), and 67 cases with high Gleason score (≥8; Table 1). A retrospective analysis of outcome assessment was done based on clinical information linked to the tissue microarray specimens. None of the patients included in this study underwent additional adjuvant therapies (e.g., hormone and radiation). Biochemical recurrence was defined as a postoperative increase in serum PSA >0.2 ng/mL. All patients had at least 1 year follow-up.

Immunohistochemistry. All antibodies were first tested and optimized on whole-tissue sections and test arrays. Once an appropriate dilution was determined, a set of three slides containing all patient samples were stained for each antibody using standard two-step indirect immunohistochemistry. After deparaffinization in xylenes, the tissue array sections were rehydrated in graded alcohols. Endogenous peroxidase was quenched with 3% hydrogen peroxide in methanol at room temperature (25°C). The sections were placed in a 95°C solution of 0.01 mol/L sodium citrate (pH 6.0) for antigen retrieval. Normal goat serum (5%) was applied for 30 min to block nonspecific protein-binding sites. Primary mouse monoclonal antibodies were applied for 30 min at room temperature at the following dilutions: TGF-β1 at 1:200 (DAKO), TβRI (TGF-β type I receptor; Santa Cruz Biotechnology) at 1:100, TβRII (TGF-β type II receptor; Upstate) at 1:200, p-Smad2 (Santa Cruz Biotechnology) at 1:400, vimentin (DAKO) at 1:500, CK18 (DAKO) at 1:500, and NF-κB at 1:200. Detection was accomplished with the DAKO Envision System followed by chromogen detection with diaminobenzidine. Sections were counterstained with Harris's hematoxylin followed by dehydration and mounting. Negative controls were done using identical array sections stained in the absence of the primary antibody. Semiquantitative assessment of all tissue microarrays was done independently by two pathologists (M.P. and X.J.Y.) who were blinded to all clinical information. The frequency of nuclear-positive cells (range, 0-100%) in prostatic glandular epithelium was scored for each tissue microarray core. Results of immunohistochemical staining were expressed as the percentage of cancer cells that stained positive for a given antibody and were scored on a scale of 0 to 3: 0 (<20% cell staining), 1 (20-49% cell staining), 2 (50-74% cell staining), and 3 (75-100% cell staining; refs. 23, 24).

Statistical analysis. Numerical data were expressed as mean ± SD. ANOVA and multiple range test were done to determine differences of means among treatment groups. $P < 0.05$ was considered as statistically significant. The SPSS 10.0.7 software package (SPSS) was used for the analysis. Kaplan-Meier survival curves were analyzed by the log-rank test using R, a statistical computing language and environment. To investigate an association with biochemical recurrence, the log-rank test was first used to determine whether various markers showed any significant effect on biochemical recurrence, which was defined as PSA > 0.2 ng/mL (25). We tested variables, including all tissue microarray markers, and TGF-β1, clinical stage, clinical (pathologic) Gleason score grouped as 4-6, 7, and 8-10, surgical margin status, and patient age. Clinical stages were divided into patients with T_1 disease or patients with $\geq T_2$ disease. Age was stratified as follows: <60, 60 to 69, and ≥70. tissue microarray Gleason score was averaged across all the tissue microarrays and divided into three: groups 4-6, 7, and 8-10. All markers were scored with a scale of 0, 1, 2, or 3 before performing recurrence analysis (23, 24).

Downloaded from clincancerres.aacrjournals.org on May 21, 2012
Copyright © 2009 American Association for Cancer Research

DOI:10.1158/1078-0432.CCR-08-1656

Table 1. Clinical characteristics of specimens for TMA

Characteristics	n (%)
No. patients	287
Age at time of surgery (y), mean (SD)	62.5 ± 8.9
Biopsy Gleason score	
4-6	62.70
7	29.30
8-10	8
Race	
White	93.70
Black	4.90
Non-Black/non-White	1.40
Clinical stage	
T_{1b}	1.00
T_{1c}	53.30
T_{2a}	24.40
T_{2b}	8.40
T_{2c}	8.00
T_{3a}	4.90
Pathologic Gleason score	
4-6	57.50
7	19.20
8-10	23.30
Positive surgical margins	20.90
Extracapsular extension	36.50
Seminal vesicle invasion	9.10
Positive lymph nodes	3.80
Perineural	53.70
Positive bone scan	8.00
Preoperative PSA (ng/mL)	
Mean (SD)	6.8 (6.6)
Median	5.5
PSA doubling time after surgery (mo)	
Median	16.1
<3	8
3-5.9	46
6-8.9	16
9-11.9	39
12-14.9	23
15-17.9	15
18-20.9	15
21-23.9	8
≥24	117

Results

NF-κB is intermediate mediator for TGF-β-induced EMT, which confers invasive potential in human prostate cancer cells. Following treatment with TGF-β1, the overall morphology of all PC-3 variants changed. We found that cells treated with TGF-β1 show elongated cell processes and have reduced numbers of cell-cell contact when compared with the untreated cells. Both changes are characteristic of EMT (26). After rendering cells insensitive to TGF-β by infection with a dominant-negative construct, TβRIIDN, or with a NF-κB inhibitor, BAY11-7085, they regained cell-cell contact and decreased the number of elongated fibroblastic processes (Fig. 1A, *left*). Western blot showed that, after infection of TβRIIDN, the activity of p-Smad2 of all PC-3, PC-3M-Pr04, PC-3M-LN4, and PC-3M was down-regulated significantly (Fig. 1B). Compared with PC-3 and PC-3M-Pr04, PC-3M and PC-3M-LN4 secreted higher baseline levels of TGF-β1 and showed increased invasive potential (Supplementary Fig. S1A). Although there was no significant difference in the original proliferation rate among PC-3 variants, PC-3 and PC-3M-Pr04 cells were less aggressive than PC-3M and PC-3M-LN4 and more sensitive to growth inhibition (>30%) by TGF-β1 by [^{3}H]thymidine incorporation assay. In contrast, PC-3M-LN4 and PC-3M were only inhibited by TGF-β1 by <7.5%. The growth-inhibitory effect of TGF-β1 disappeared when these cells were rendered insensitive to TGF-β by infection with TβRIIDN (Supplementary Fig. S1B).

There was a dose-dependent relationship between TGF-β1 exposure and the expression of vimentin (positive correlation) and CK18 (negative correlation; Fig. 1C). Untreated PC-3M and PC-3M-LN4 cells constitutively express high levels of vimentin but down-regulated or devoid of CK18 expression in comparison with PC-3. In contrast, the untreated PC-3 and PC-3M-Pr04 expressed relatively low levels of vimentin and relatively high levels of CK18 (Fig. 1D; Supplementary Fig. S2). In addition, the level of NF-κB expression in these cells was significantly increased following treatment with TGF-β1 (Fig. 2). However, following transfection with TβRIIDN, the level of vimentin expression (Fig. 1D) and NF-κB (Fig. 2) was significantly reduced, whereas CK18 increased simultaneously. This was observed in all cell lines, except for PC-3M-LN4, the most aggressive among the PC-3 variants. Interestingly, on treatment with NF-κB inhibitor, the effect of TGF-β1 on EMT was abrogated (Fig. 1D). The role of NF-κB in TGF-β-induced EMT was further supported by coexpression of vimentin and NF-κB in all PC-3 cell lines. Only those cells expressing NF-κB showed expression of vimentin. As expected, when cells were transfected by TβRIIDN, the level of expression of both vimentin and NF-κB was reduced significantly (Fig. 3A).

Using a Transwell chamber assay, before TGF-β1 treatment, PC-3M-LN4 and PC-3M had a higher invasive potential than that for PC-3 and PC-3M-Pr04 (Fig. 3B-D), which may due to the higher baseline level of TGF-β1 secreted by PC-3M-LN4 and PC-3M (Fig. 1B, *top*). With the treatment of TGF-β1, there was a further increase in invasion in each group. Invasion of all PC-3 variants was inhibited by transfection with TβRIIDN or by NF-κB inhibitor. Importantly, the inhibition of invasion by the NF-κB inhibitor could not be reversed by TGF-β1 treatment (Fig. 3B-D). This observation suggested that NF-κB was downstream of TGF-β, and NF-κB and TGF-β could synergistically mediate EMT and invasion in PC-3 cells. Taken together, these results are consistent with the notion that NF-κB is a mediator for the TGF-β-induced EMT, leading to an invasive phenotype in PC-3 variants.

TGF-β-induced EMT correlates with clinical characteristics. To evaluate the association of TGF-β with the induction of the EMT in prostate cancer specimens, we performed immunohistochemistry on tissue microarrays. Expression levels of TGF-β signaling components including TGF-β1, TβRI, TβRII, p-Smad2, and EMT-associated factors, such as vimentin, CK18, and NF-κB, were determined using specific antibodies. Using tissue microarray specimens, we found that a high level of expression of TGF-β1 and EMT-related factors coupled with a low level of expression of TβRI, TβRII, and p-Smad2 was associated with adverse pathologic features, such as increased Gleason grade (Fig. 4A). For instance, high levels of TGF-β1 expression (75-100% positively stained cancer cells) were identified in 36.7%, 6.7%, and 6.0% for tumors with high (≥8), intermediate (7), and low (≤6) Gleason scores, respectively. High levels of NF-κB expression were found in 60.6%, 10%, and 5.2%, vimentin expression in 23.9%, 3.6%, and 1.8%,

Downloaded from clincancerres.aacrjournals.org on May 21, 2012
Copyright © 2009 American Association for Cancer Research

DOI:10.1158/1078-0432.CCR-08-1656

and CK18 expression in 47.1%, 67.2%, and 68% of high, intermediate, and low Gleason score cancers, respectively (Fig. 4B). TGF-β signaling components were directly correlated with increasing Gleason grade. The regression coefficient (R) and the significance level (P) for these parameters were as follows: TGF-β1 (R = 0.423, P = 1.152411e-13), NF-κB (R = 0.512, P = 2.2e-16), and vimentin (R = 0.375, P = 1.45e-10). TβRI (R = -0.285, P = 1.14), TβRII (R = -0.14, P = 0.019), p-Smad2 (R = -0.181, P = 0.034), and CK18 (R = -0.221, P = 0.015) were negatively correlated with increasing Gleason score (Supplementary Table S1). Correspondingly, we found decreased expression of TβRI and TβRII in PC-3M and PC-3M-LN4, which are more aggressive phenotypes, compared with PC-3 and PC-3M-Pr04, which are less aggressive phenotypes (Supplementary Fig. S3). Because of a relatively low degree of correlation between the level of TGF-β1 expression and that of p-Smad2 activity but a relatively high degree of correlation between TGF-β1 and expression of EMT factors, we postulated that a Smad-independent pathway was probably responsible for the TGF-β-induced EMT.

Using tissue microarray specimens, we also found a relationship between levels of expression of TGF-β1 and EMT-related factors. In all tissue microarrays analyzed, there was a significant correlation between the expression of TGF-β1 and vimentin (R = 0.284, P = 2.38e-06), TGF-β1 and NF-κB (R = 0.313, P = 4.27e-07), vimentin and NF-κB (R = 0.429, P = 3.04e-12), and vimentin and CK18 (R = -0.164, P = 0.010; Supplementary Table S1). Taken together, these results suggest that the level of TGF-β expression is significantly associated with the expression levels of NF-κB and many other EMT-associated proteins.

We found that there was a correlation between age and expression of TGF-β1 (R = 0.204, P = 0.002206915) and CK18 (R = -0.305, P = 9.981004e-06), respectively. Unexpectedly, we found a positive correlation between patient age and expression of TGF-β1 (R = 0.204, P = 0.002) but a negative correlation between patient age and expression of CK18 (R = -0.305, P = 9.98e-06). Prostate cancer specimens of older patients (ages ≥70 years) showed a higher level of expression of TGF-β1 but a lower level of CK18, a condition conducive for EMT induction. Another finding of interest was that levels of expression of the above tumor markers correlated significantly with the percentage of cancer in the prostate, which was calculated using the total amount of tumor in the frozen tissue as the percentage of the entire prostate. The expression level of TGF-β1 (R = 0.295, P = 0.007), NF-κB (R = 0.393, P = 0.001), vimentin (R = 0.445, P = 3.19e-05), and CK18 (R = -0.362, P = 0.003) in the tumor all correlated significantly with the percentage of cancer (Supplementary Table S1). These data support the concept the induction of EMT is TGF-β-dependent but Smad-independent in prostate cancer.

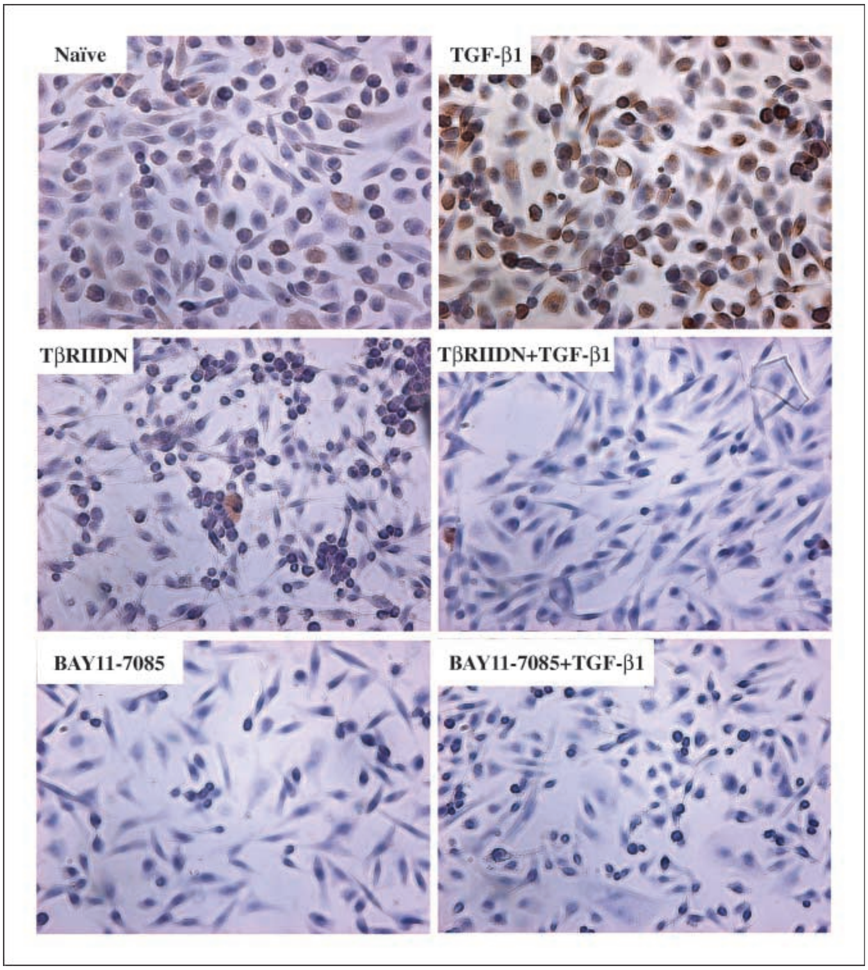

Fig. 2. TGF-β induces the expression of NF-κB in prostate cancer. Immunohistochemistry for NF-κB in all cell lines shows that, under untreated conditions, cancer cells express some NF-κB (with PC-3 as a sample, the confluent percentage is 95%). However, expression of NF-κB is up-regulated significantly after treatment with TGF-β (10 ng/mL, 24 h). Meanwhile, in prostate cancer cells rendered insensitive to TGF-β (e.g., expression of TβRIIDN or treatment with BAY11-7085), expression of NF-κB was inhibited dramatically even after addition of exogenous TGF-β (magnification, 10 × 20).

Downloaded from clincancerres.aacrjournals.org on May 21, 2012
Copyright © 2009 American Association for Cancer Research

DOI:10.1158/1078-0432.CCR-08-1656

NF-κB-mediated TGF-β-induced EMT predicts biochemical recurrence in prostate cancer patients. We next compared the clinical outcomes to the above-mentioned markers. Expression levels of TGF-β1 (P = 0.0158), vimentin (P = 0.004), CK18 (P = 0.006), NF-κB (P = 0.030), patient age (P = 1.99e-05), pathologic stage (P = 6.5e-07), and clinical Gleason score (P = 0.005) all correlated significantly with biochemical recurrence, defined as a PSA >0.2 ng/mL after radical prostatectomy (Fig. 5A; Supplementary Table S2). However, clinical stage, surgical margin status, and expression of TβRI, TβRII, and p-Smad2 did not correlate with biochemical recurrence (P > 0.05). A Kaplan-Meier curve was generated for each of the above significant variables. To determine the best model for predicting biochemical recurrence, Cox proportional hazards model was fit to include all the significant variables (Fig. 5A) and backward selection method was used to eliminate nonsignificant variables. The final selected model included vimentin, grouped as scale <3 or 3 (log-rank P = 0.049; hazard ratio, 2.03; 95% confidence interval, 1-4.12), and pathologic stage grouped as $<T_{3a}$ and $\geq T_{3a}$ (log-rank P = 0.031; hazard ratio, 10.1; 95% confidence interval, 1.23-82.8; Fig. 5B and C; Supplementary Table S3). Tumors with a vimentin expression score of 3 had a 2.03 higher risk of recurrence than patients with lower scores of vimentin

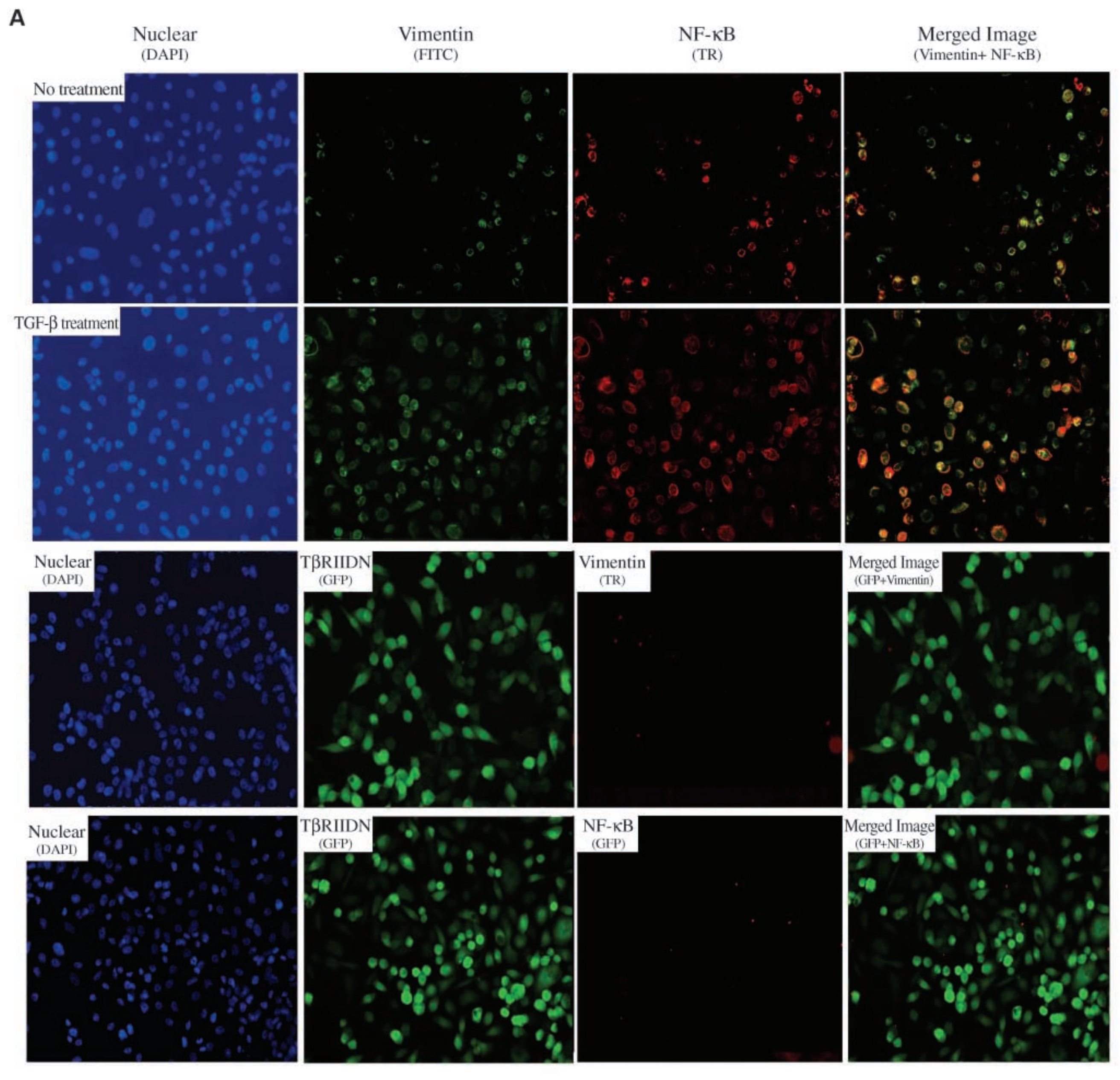

Fig. 3. NF-κB-mediated TGF-β1-induced EMT is the cause of invasion of prostate cancer. *A,* colocalization of TGF-β signal and NF-κB and vimentin were analyzed in all cell lines by using immunofluorescein staining (with PC-3 as sample). Only the cells expressing NF-κB exhibited vimentin expression. In control, after cells were infected with TβRIIDN, both vimentin and NF-κB were inhibited dramatically (magnification, 10 × 20).

Downloaded from clincancerres.aacrjournals.org on May 21, 2012
Copyright © 2009 American Association for Cancer Research

DOI:10.1158/1078-0432.CCR-08-1656

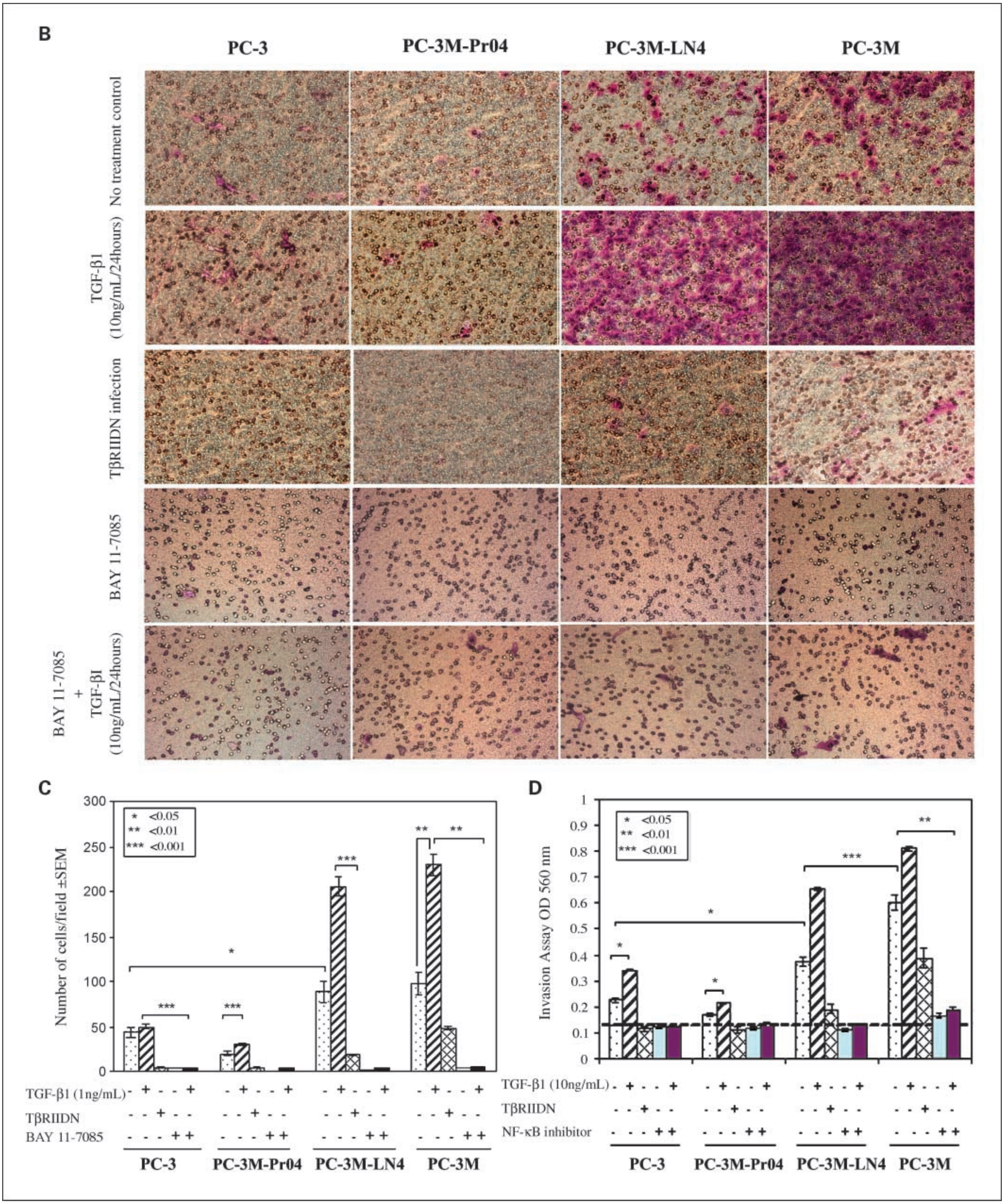

Fig. 3 *Continued. B,* PC-3M-LN4 and PC-3M possessed significantly higher invasive capabilities when compared with PC-3 and PC-3M-Pr04. There was a significant increase in cell motility through a Matrigel-coated polycarbonate membrane under the treatment of TGF-β1 (10 ng/mL). The invasion of all PC-3 cells could be inhibited by blocking the TGF-β signal by infection with a TβRIIDN or using a NF-κB inhibitor separately. The inhibition of invasion by NF-κB is reverted by TGF-β treatment. *C,* corresponding numbers of invasive cells. *D,* absorbance values. This result indicates NF-κB-mediated TGF-β-induced EMT potentiates the invasive ability of prostate cancer cell lines.

Downloaded from clincancerres.aacrjournals.org on May 21, 2012
Copyright © 2009 American Association for Cancer Research

DOI:10.1158/1078-0432.CCR-08-1656

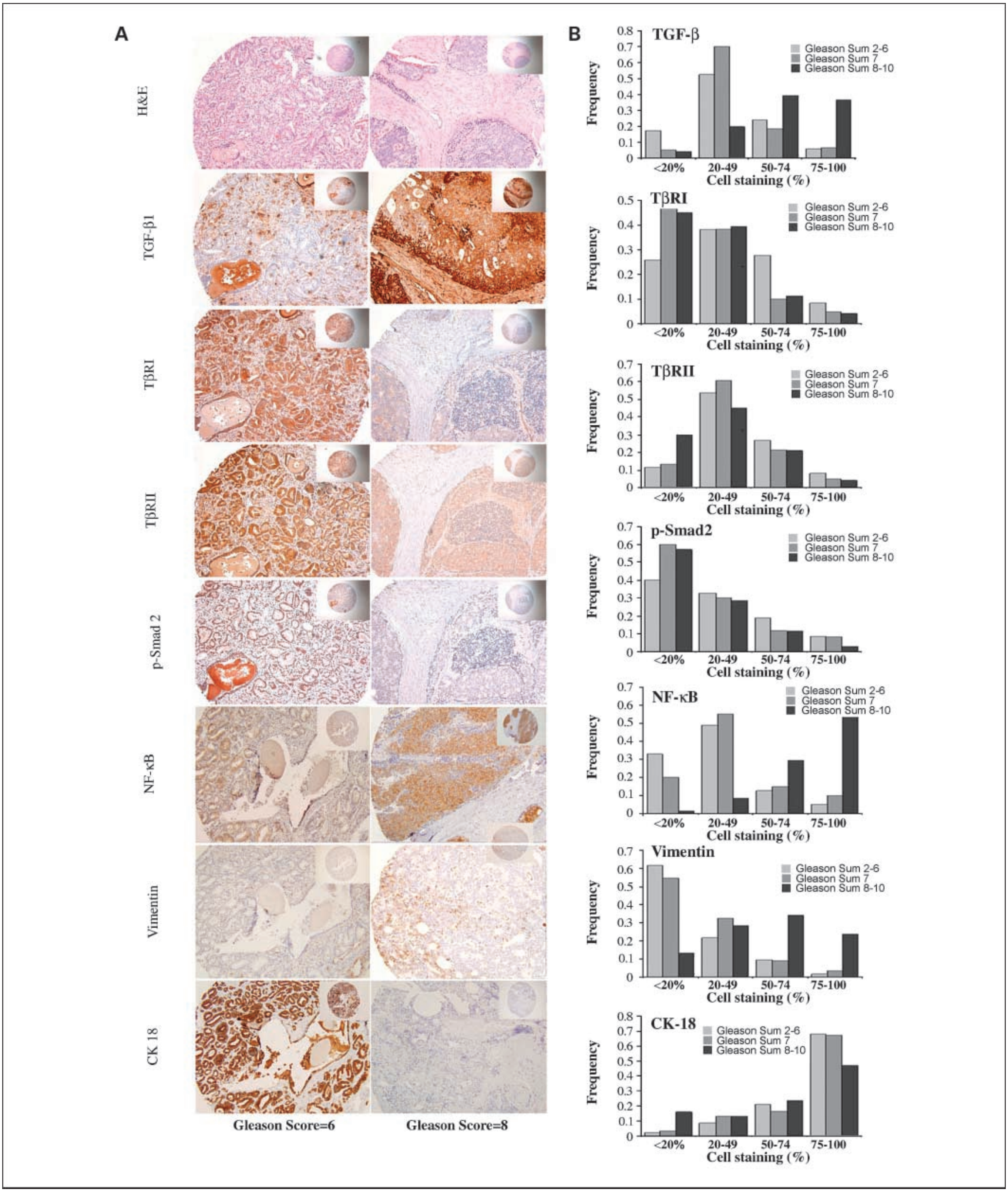

Fig. 4. NF-κB-mediated TGF-β1-induced EMT is determined by tissue microarrays and immunohistochemistry. *A,* in serial tissue microarray sections from a patient with Gleason score of 8, staining revealed higher expression of TGF-β, NF-κB, and vimentin but lower expression of TGF-β receptors and p-Smad2 compared with serial sections taken from a patient with a lower Gleason grade of 6. Representative of the predominant staining pattern seen in all patient samples/tissue arrays (magnification, 10 × 20). *B,* corresponding frequency (or percentage) of staining and intensity of staining. High levels of TGF-β1 expression (75-100% cell staining) were identified in 36.7%, 6.7%, and 6.0% of high-grade, intermediate-grade, and low-grade prostate cancers, respectively. The expression of NF-κB was 60.6%, 10%, and 5.2% and vimentin expression was 23.9%, 3.6%, and 1.8% in high-grade, intermediate-grade, and low-grade prostate cancers, respectively. CK18 was 47.1%, 67.2%, and 68% in high-grade, intermediate-grade, and low-grade prostate cancers, respectively.

Downloaded from clincancerres.aacrjournals.org on May 21, 2012
Copyright © 2009 American Association for Cancer Research

DOI:10.1158/1078-0432.CCR-08-1656

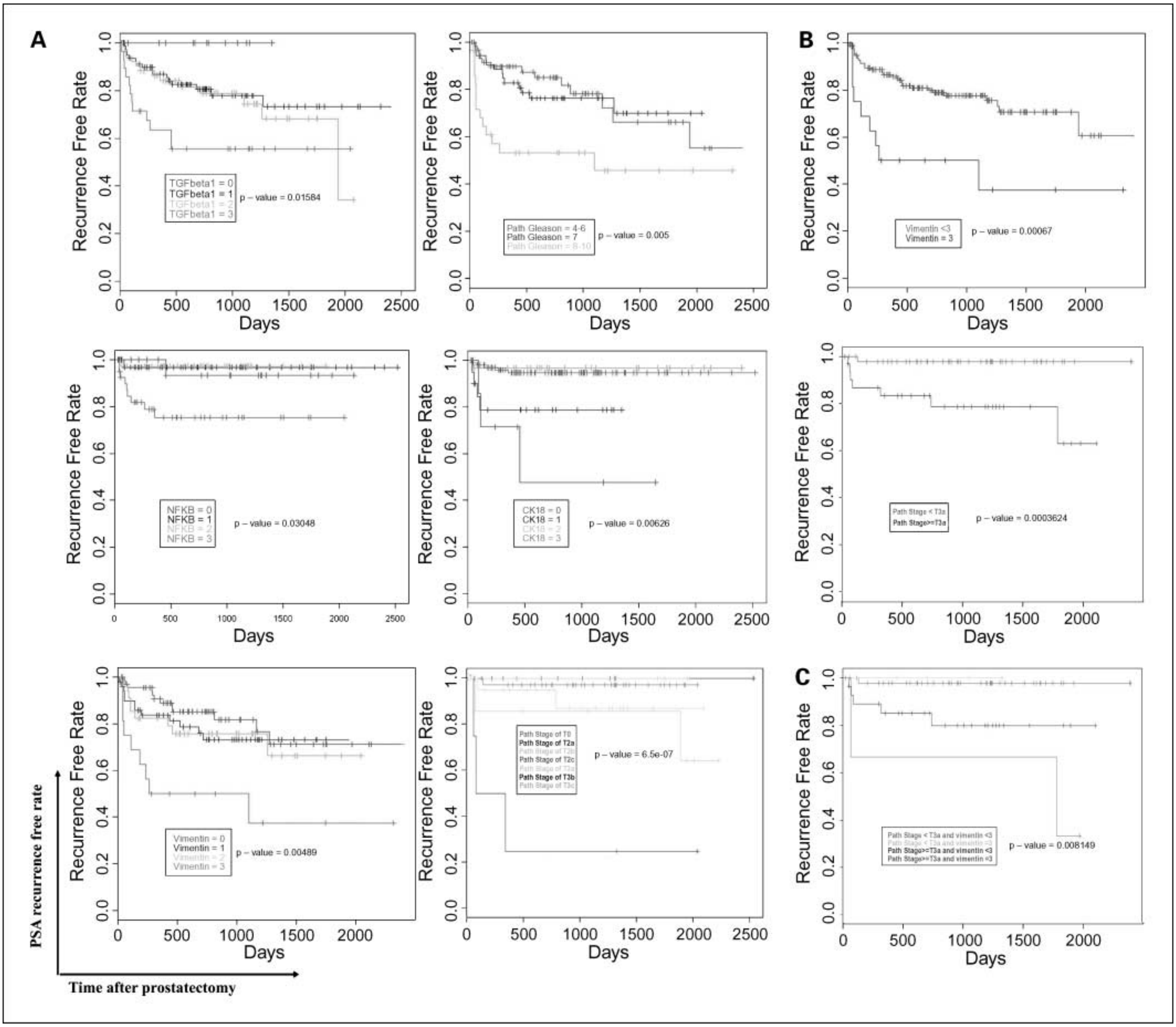

Fig. 5. NF-κB-mediated TGF-β-induced EMT predicts prostate cancer recurrence. *A,* Kaplan-Meier curve was generated for significant variables. TGF-β1 ($P = 0.0158$), vimentin ($P = 0.00489$), CK18 ($P = 0.00626$), NF-κB ($P = 0.0305$), pathologic stage ($P = 6.5e\text{-}07$), and Gleason score ($P = 0.005$) all have significant effect on PSA recurrence. On the other hand, clinical stage, surgical margin status, TβRI, TβRII, and p-Smad2 had no significant effect on PSA recurrence ($P > 0.05$). *B,* vimentin expression was classified into two groups (3 and <3) and pathologic stage as T_0, T_1, T_2, and T_3 with scores 0, 1, 2, and 3, respectively. There was a significant difference in the survival curves. *C,* Cox proportional hazards model was fit to include all the significant variables and backward selection method was used to eliminate nonsignificant variables and the final selected model includes vimentin (log-rank $P = 0.049$; hazard ratio, 2.03; 95% confidence interval, 1-4.12) grouped as <3 and 3 and pathologic stage grouped as $<T_{3a}$ and $\geq T_{3a}$ (log-rank $P = 0.031$; hazard ratio, 10.1; 95% confidence interval, 1.23-82.8). Patients with pathologic stage $>T_{3a}$ have a 10.1 times higher biochemical recurrence rate than patients with pathologic stage underwent T_{3a}. Patients with tissue level vimentin of 3 had a 2.03 times higher biochemical recurrence rate than patients with lower tissue levels of vimentin.

expression in the tumor. There was a high risk of recurrence if high levels of expression of TGF-β1, vimentin, and NF-κB and low level of CK18 were noted in the tumor. This was particularly true for vimentin, which is independent of patients' Gleason score. These findings suggest that proteins associated with the EMT predict biochemical recurrence in prostate cancer patients. Clinical Gleason score did not add any predictive value to the proportional hazards model despite that clinical Gleason score sum alone had a significant effect on biochemical recurrence. Also, we did not find any correlation between PSA doubling time with the level of expression of EMT markers, such as TGF-β1, vimentin, and NF-κB. Taken together, markers of the EMT were stronger predictors of PSA recurrence than variables such as clinical Gleason score and PSA doubling time.

Discussion

Results of the present study suggest that TGF-β can mediate the EMT in prostate cancer cells, which results in an aggressive phenotype. This is possibly mediated by NF-κB, which is

Downloaded from clincancerres.aacrjournals.org on May 21, 2012
Copyright © 2009 American Association for Cancer Research

DOI:10.1158/1078-0432.CCR-08-1656

considered to be an essential factor for EMT induction (16, 18, 27–30). Ao et al. have proposed that TGF-β promotes invasion in tumorigenic, but not nontumorigenic, prostatic epithelial cells and that TGF-β regulation of vimentin expression is dependent on Akt (15). In the present study, we have confirmed that NF-κB is an integral downstream factor of TGF-β-induced EMT. Because Akt is known to promote NF-κB activity via IKKs (11), in combination with the Ao et al. study, we connected this downstream effector between TGF-β-induced EMT in prostate cancer. More importantly, we found that the detection of EMT in tumor specimens obtained at the time of radical prostatectomy may serve as a predictor of biochemical recurrence in prostate cancer patients following radical prostatectomy.

Transition of cells from an epithelial to a mesenchymal phenotype confers a loss of sensitivity to growth inhibition by TGF-β and manifests an invasive and metastatic phenotype (31). Our results show that TGF-β inhibits prostate cancer cells with a less aggressive phenotype, such as PC-3 and PC-3M-Pr04, but not with the highly aggressive cancer cells, such as PC-3M-LN4 and PC-3M. These latter two cell lines have escaped the growth inhibition by TGF-β, which is linked to a strong induction of EMT and a reduced expression of TGF-β receptors. For example, we observed similar trends in specimens obtained. High levels of expression of TGF-β, vimentin, and NF-κB were identified in 36.7%, 66.6%, and 23.9% of tumors with high, intermediate, and low Gleason grades, respectively. Furthermore, in tumors with intermediate or low Gleason grade, the percentage of positively stained cancer cells for these markers was relatively reduced. There is a significant positive correlation between expression of TGF-β1 and expression of EMT-related factors such as vimentin, NF-κB, and CK18. Consistent with observations obtained from cell lines, TGF-β is a mediator for EMT in prostate cancer.

In the present study, low levels of p-Smad2 were found in tumors with high Gleason score. This finding suggests that these tumors have escaped the growth inhibition by TGF-β. The signaling pathways in TGF-β-induced EMT can be divided into Smad-independent (32–36) and Smad-dependent (37) pathways. Recent reports indicated that phosphorylation of Smad3 at the linker region may be involved in the EMT, which is TβRI and p-Smad2 independent (31, 37). Results of the present study showed that, in prostate cancer, TGF-β, rather than Smads, mediated the activation of NF-κB. Furthermore, we have shown NF-κB to be the major downstream mediator of TGF-β-induced EMT in prostate cancer cells. Our results indicate that blockade of either TGF-β or NF-κB reverses the EMT process. Overexpression of TGF-β1 by tumor cells will result in the induction of NF-κB and vimentin expression, which confers an aggressive phenotype. Although other factors such as Ras, Jag1, and Hey1 may be involved in the process (17, 18), NF-κB is an obligatory switch for TGF-β-induced EMT in prostate cancer.

The present results indicate that TGF-β-induced EMT correlates with tumor invasiveness and biochemical recurrence in prostate cancer patients. NF-κB is the essential intermediate mediator for TGF-β-induced EMT in prostate cancer. Within 7 years following radical prostatectomy, variables including TGF-β1, NF-κB, vimentin, CK18, and patient age correlated significantly with biochemical recurrence. We confirmed that high stage is associated with higher recurrence rate than those undergone surgery at a lower pathologic stage (38). We show that patients with a higher tissue level of vimentin have higher biochemical recurrence rate than those with a lower tissue level of vimentin. Furthermore, the relationship between vimentin expression and biochemical recurrence appears to be independent of pathologic stage or PSA doubling time. This indicates that a high level of TGF-β-induced expression of vimentin (scale 3: 75-100% positively stained cancer cells) in prostatectomy specimens may be used to predict biochemical recurrence in prostate cancer patients. Our result indicates that vimentin in combination with pathologic stage are good predictors for disease outcome. The exact mechanism of this observation remains unclear, but tumors from older patients have higher expression levels of TGF-β1 and lower levels of CK18 expression than those of their younger counterparts, which indicated that EMTs are more likely to occur in former group. Based on our results, during progression of prostate cancer, an attenuation of expression of TGF-β receptors facilitates tumor cells escaping from the growth inhibition by TGF-β, which is Smad dependent. Meanwhile, the Smad-independent pathway, such as NF-κB signaling, mediates the EMT process, which could be reversed by the blockade of TGF-β signaling by infection with TβRIIDN or by the use of the NF-κB inhibitor. Variables such as tumor expression of EMT markers and TGF-β1, or age of patients, may be useful in predicting clinical outcome following radical prostatectomy. High levels of vimentin (75-100% positively stained cancer cells) and pathologic stage ($\geq T_{3a}$) proved to be risk factors for biochemical recurrence in prostate cancer patients regardless of clinical Gleason score.

In summary, our findings have indicated that NF-κB-mediated TGF-β-induced EMT may be used to predict patients at high risk for biochemical recurrence following radical prostatectomy. This event is independent of Gleason score or PSA doubling time. Transition of cancer cells from an epithelial to a mesenchymal phenotype induces loss of sensitivity to growth inhibition by TGF-β and confers an invasive phenotype (31). It is conceivable that the EMT-related factors may be used as prognostic markers for other types of cancer.

Disclosure of Potential Conflicts of Interest

No potential conflicts of interest were disclosed.

References

1. Jemal A, Siegel R, Ward E, Murray T, Xu J, Thun MJ. Cancer statistics, 2007. CA Cancer J Clin 2007;57:43–66.
2. Walsh PC, Retik AB, Vaughan ED, et al. Campbell's urology. 7th ed. Philadelphia (PA): WB Saunders; 1998.
3. Roehl KA, Han M, Ramos CG, Antenor JA, Catalona WJ. Cancer progression and survival rates following anatomical radical retropubic prostatectomy in 3,478 consecutive patients: long-term results. J Urol 2004;172:910–4.
4. Stephenson AJ, Kattan MW, Eastham JA, et al. Defining biochemical recurrence of prostate cancer after radical prostatectomy: a proposal for a standardized definition. J Clin Oncol 2006;24:3973–8.
5. Freedland SJ, Humphreys EB, Mangold LA, et al. Risk of prostate cancer-specific mortality following biochemical recurrence after radical prostatectomy. JAMA 2005;294:433–9.
6. Phillips C. Studies shed more light on value of

Downloaded from clincancerres.aacrjournals.org on May 21, 2012
Copyright © 2009 American Association for Cancer Research

DOI:10.1158/1078-0432.CCR-08-1656

"PSA kinetics" in prostate cancer. Natl Cancer Inst Can Bull 2005;32:3.

7. Grizzle WE, Myers RB, Manne U, Srivastava S. Immunohistochemical evaluation of biomarkers in prostate and colorectal neoplasia: principles and guidelines. In: Hanausek M, Walaszek Z, editors. Methods in molecular medicine. Vol. 14. Tumor marker protocols. Totowa (NJ): Humana Press; 1998. p. 143–60.
8. Gleason DF. Classification of prostatic carcinomas. Cancer Chemother Rep 1966;50:125–8.
9. Bunting PS. Screening for prostate cancer with prostate-specific antigen: beware the biases. Clin Chim Acta 2002;315:71–97.
10. Thiery JP. Epithelial-mesenchymal transitions in tumour progression. Nat Rev Cancer 2002;2:442–54.
11. Thiery JP, Sleeman JP. Complex networks orchestrate epithelial-mesenchymal transitions. Nat Rev Mol Cell Biol 2006;7:131–42.
12. Bierie B, Moses HL. Tumour microenvironment: TGFβ: the molecular Jekyll and Hyde of cancer. Nat Rev Cancer 2006;6:506–20.
13. Wei J, Xu G, Wu M, et al. Overexpression of vimentin contributes to prostate cancer invasion and metastasis via src regulation. Anticancer Res 2008;28:327–34.
14. Lang SH, Hyde C, Reid IN, et al. Enhanced expression of vimentin in motile prostate cell lines and in poorly differentiated and metastatic prostate carcinoma. Prostate 2002;52:253–63.
15. Ao M, Williams K, Bhowmick NA, Hayward SW. Cross-talk between paracrine-acting cytokine and chemokine pathways promotes malignancy in benign human prostatic epithelium. Cancer Res 2007;67:4244–53.
16. Zavadil J, Bottinger EP. TGF-β and epithelial-to-mesenchymal transitions. Oncogene 2005;24:5764–74.
17. Han G, Lu SL, Li AG, et al. Distinct mechanisms of TGF-β1-mediated epithelial-to-mesenchymal transition and metastasis during skin carcinogenesis. J Clin Invest 2005;115:1714–23.
18. Huber MA, Azoitei N, Baumann B, et al. NF-κB is essential for epithelial-mesenchymal transition and metastasis in a model of breast cancer progression. J Clin Invest 2004;114:569–81.
19. Pettaway CA, Pathak S, Greene G, et al. Selection of highly metastatic variants of different human prostatic carcinomas using orthotopic implantation in nude mice. Clin Cancer Res 1996;2:1627–36.
20. Zhang Q, Yang X, Pins M, et al. Adoptive transfer of tumor-reactive transforming growth factor-β-insensitive CD8+ T cells: eradication of autologous mouse prostate cancer. Cancer Res 2005;65:1761–9.
21. Zhang Q, Yang XJ, Kundu SD, et al. Blockade of transforming growth factor-β signaling in tumor-reactive CD8(+) T cells activates the antitumor immune response cycle. Mol Cancer Ther 2006;5:1733–43.
22. Zhang Q, Rubenstein JN, Jang TL, et al. Insensitivity to transforming growth factor-β results from promoter methylation of cognate receptors in human prostate cancer cells (LNCaP). Mol Endocrinol 2005;19:2390–9.
23. Steg A, Vickers SM, Eloubeidi M, et al. Hedgehog pathway expression in heterogeneous pancreatic adenocarcinoma: implications for the molecular analysis of clinically available biopsies. Diagn Mol Pathol 2007;16:229–37.
24. Grizzle WE, Myers RB, Manne U, Srivastava S. Immunohistochemical evaluation of biomarkers in prostate and colorectal neoplasia: principles and guidelines. In: Hanausek M, Walaszek Z, editors. Methods in molecular medicine. Vol. 14. Tumor marker protocols. Totowa (NJ): Humana Press; 1998. p. 143–60.
25. Cookson MS, Aus G, Burnett AL, et al. Variation in the definition of biochemical recurrence in patients treated for localized prostate cancer: the American Urological Association Prostate guidelines for localized prostate cancer update panel report and recommendations for a standard in the reporting of surgical outcomes. J Urol 2007;177:540–5.
26. Lee JM, Dedhar S, Kalluri R, Thompson EW. The epithelial-mesenchymal transition: new insights in signaling, development, and disease. J Cell Biol 2006;172:973–81.
27. Park JI, Lee MG, Cho K, et al. Transforming growth factor-β1 activates interleukin-6 expression in prostate cancer cells through the synergistic collaboration of the Smad2, p38-NF-κB, JNK, and Ras signaling pathways. Oncogene 2003;22:4314–32.
28. Yang J, Mani SA, Donaher JL, et al. Twist, a master regulator of morphogenesis, plays an essential role in tumor metastasis. Cell 2004;117:927–39.
29. Kang Y, Massague J. Epithelial-mesenchymal transitions: twist in development and metastasis. Cell 2004;118:277–9.
30. Huber MA, Beug H, Wirth T. Epithelial-mesenchymal transition: NF-κB takes center stage. Cell Cycle 2004;3:1477–80.
31. Matsuzaki K. Smad3 phosphoisoform-mediated signaling during sporadic human colorectal carcinogenesis. Histol Histopathol 2006;21:645–62.
32. Bakin AV, Tomlinson AK, Bhowmick NA, Moses HL, Arteaga CL. Phosphatidylinositol 3-kinase function is required for transforming growth factor β-mediated epithelial to mesenchymal transition and cell migration. J Biol Chem 2000;275:36803–10.
33. Bhowmick NA, Ghiassi M, Bakin A, et al. Transforming growth factor-β1 mediates epithelial to mesenchymal transdifferentiation through a RhoA-dependent mechanism. Mol Biol Cell 2001;12:27–36.
34. Janda E, Lehmann K, Killisch I, et al. Ras and TGFβ cooperatively regulate epithelial cell plasticity and metastasis: dissection of Ras signaling pathways. J Cell Biol 2002;156:299–313.
35. Yu L, Hebert MC, Zhang YE. TGF-β receptor-activated p38 MAP kinase mediates Smad-independent TGF-β responses. EMBO J 2002;21:3749–59.
36. Dumont N, Bakin AV, Arteaga CL. Autocrine transforming growth factor-β signaling mediates Smad-independent motility in human cancer cells. J Biol Chem 2003;278:3275–85.
37. Saika S, Kono-Saika S, Ohnishi Y, et al. Smad3 signaling is required for epithelial-mesenchymal transition of lens epithelium after injury. Am J Pathol 2004;164:651–63.
38. Magheli A, Rais-Bahrami S, Trock BJ, et al. Impact of body mass index on biochemical recurrence rates after radical prostatectomy: an analysis utilizing propensity score matching. Urology. Epub 2008.

Downloaded from clincancerres.aacrjournals.org on May 21, 2012
Copyright © 2009 American Association for Cancer Research
